# XAF1 Protects Host against Emerging RNA Viruses by Stabilizing IRF1-Dependent Antiviral Immunity

**DOI:** 10.1128/jvi.00774-22

**Published:** 2022-08-16

**Authors:** Yu Han, Xue Bai, Siying Liu, Jingfei Zhu, Fan Zhang, Lifen Xie, Guodi Liu, Xiaohui Jiang, Mingchao Zhang, Yingkang Huang, Jingfeng Wang, Dapei Li, Huiying Zhang, Yuanqing He, Sudan He, Yu Xia, Xiulong Xu, Feng Xu, Feng Ma

**Affiliations:** a Department of Infectious Diseases, The Second Affiliated Hospital of Zhejiang University School of Medicine, Hangzhou, China; b CAMS Key Laboratory of Synthetic Biology Regulatory Elements, Institute of Systems Medicine, Chinese Academy of Medical Sciences & Peking Union Medical College, Beijing, China; c Suzhou Institute of Systems Medicine, Suzhou, China; d Institute of Comparative Medicine, College of Veterinary Medicine, Yangzhou University, Yangzhou, China; e Suzhou Center for Disease Control and Prevention, Suzhou, China; University of North Carolina at Chapel Hill

**Keywords:** XAF1, IRF1, antiviral immunity, type I interferon, ISG, ubiquitination

## Abstract

XIAP-associated factor 1 (XAF1) is an interferon (IFN)-stimulated gene (ISG) that enhances IFN-induced apoptosis. However, it is unexplored whether XAF1 is essential for the host fighting against invaded viruses. Here, we find that XAF1 is significantly upregulated in the host cells infected with emerging RNA viruses, including influenza, Zika virus (ZIKV), and SARS-CoV-2. IFN regulatory factor 1 (IRF1), a key transcription factor in immune cells, determines the induction of XAF1 during antiviral immunity. Ectopic expression of XAF1 protects host cells against various RNA viruses independent of apoptosis. Knockout of XAF1 attenuates host antiviral innate immunity *in vitro* and *in vivo*, which leads to more severe lung injuries and higher mortality in the influenza infection mouse model. XAF1 stabilizes IRF1 protein by antagonizing the CHIP-mediated degradation of IRF1, thus inducing more antiviral IRF1 target genes, including *DDX58*, *DDX60*, *MX1*, and *OAS2*. Our study has described a protective role of XAF1 in the host antiviral innate immunity against RNA viruses. We have also elucidated the molecular mechanism that IRF1 and XAF1 form a positive feedback loop to induce rapid and robust antiviral immunity.

**IMPORTANCE** Rapid and robust induction of antiviral genes is essential for the host to clear the invaded viruses. In addition to the IRF3/7-IFN-I-STAT1 signaling axis, the XAF1-IRF1 positive feedback loop synergistically or independently drives the transcription of antiviral genes. Moreover, XAF1 is a sensitive and reliable gene that positively correlates with the viral infection, suggesting that XAF1 is a potential diagnostic marker for viral infectious diseases. In addition to the antitumor role, our study has shown that XAF1 is essential for antiviral immunity. XAF1 is not only a proapoptotic ISG, but it also stabilizes the master transcription factor IRF1 to induce antiviral genes. IRF1 directly binds to the IRF-Es of its target gene promoters and drives their transcriptions, which suggests a unique role of the XAF1-IRF1 loop in antiviral innate immunity, particularly in the host defect of IFN-I signaling such as invertebrates.

## INTRODUCTION

Innate antiviral immunity is the first line of host defense against pathogenic viruses. Emerging and reemerging viruses such as influenza H7N9, MERS-CoV, Zika virus (ZIKV), and SARS-CoV-2 outbroke worldwide and have infected more than 400 million people and killed over 5 million people in the past several years (1–3). During infection by these life-threatening viruses, viral RNAs are recognized by pattern recognition receptors (PRRs) to initiate complex signal transduction pathways, which leads to the induction of type I interferon (IFN-I) and proinflammatory cytokines (4–6). Retinoic acid-inducible gene I (RIG-I), also known as DDX58, is one of the most important cytosolic RNA sensors that recognizes viral RNA derived from invaded viruses (6, 7). Activating RIG-I by viral RNA results in the phosphorylation of TBK1 and IRF3, which activates transcription factors (TFs) including NF-κB, AP-1, and IRF3/7 to induce proinflammatory cytokines and IFN-I (8). IFN-I, including IFN-α and IFN-β, further triggers the phosphorylation of TFs STAT1 and STAT2 via the JAK-STAT pathway to induce over 300 IFN-stimulated genes (ISGs), which synergistically inhibit viral infection by targeting almost all the steps of viral life cycles (9, 10). For example, MX1 is a GTPase that inhibits viral infection by blocking viral transcription, replication, and assembly (9, 11). The 2′,5′-oligoadenylate synthetase 2 (OAS2) binds and activates RNase L to degrade viral RNA in the cytoplasm of host cells and eventually prevent protein synthesis and viral RNA replication (9). DDX60, a DEXD/H box helicase, is a novel antiviral factor that facilitates RIG-I-like receptor-mediated signaling and XRN1 RNA 5′–3′ exonuclease-dependent viral RNA degradation (12, 13). However, the functions of many other ISGs in host innate immunity against viral infection are still unexplored.

X-linked inhibitor of apoptosis (XIAP)-associated factor 1 (XAF1) is identified as an ISG (14–16), which mainly mediates the IFN-β-TRAIL-induced apoptosis in multiple cell lines and functions as a tumor suppressor (14, 17–19). The XAF1 protein contains seven tumor TNF receptor-related factor (TRAF)-like zinc finger (ZF) domains, which interact with IFN regulatory factor 1 (IRF1) and p53 under stress conditions (17, 19). XAF1 suppresses tumorigenesis by inducing the transcripts of p53 and stabilizing p53 protein (17). Genetic mutation of XAF1 determines p53 expression and cancer susceptibility (20). In addition to the important roles of XAF1 as a proapoptotic factor in cancer, it is unknown whether XAF1 is also involved in the regulation of IFN-I-triggered antiviral immunity, as multiple antiviral ISGs do during viral infection. It has been reported that expression of XAF1 is induced during dengue virus (DENV) infection, and XAF1 contributes to DENV-triggered apoptosis in vascular endothelial cells (21). Despite the potential correlation between XAF1 and viral infection, the function of XAF1 in the hosts fight against viral infection is unclear.

IRF1, the first member of the IRF family, was originally identified as a nuclear factor that binds and activates the promoters of IFN-I genes (22, 23). IRF1 is also required for IFN-l induction to combat MERS-CoV infection in human epithelial cells and *in vivo* (24). The STAT1-IRF1-NLRC5 axis plays a critical role in the MHC class I-mediated antigen presentation pathway during SARS-CoV-2 infection (25). IRF1 is unique and important in innate immunity against pathogens, particularly RNA viruses. Unlike other IRFs, IRF1 exists in a wide swath of invertebrates and represents one of the most ancient components of innate immunity (26). IRF1 is also evolutionally conserved in multiple vertebrates and identified as one of the key TFs included in a core group of 62 ISGs shared by 9 different mammalian species, including bats, the hosts for many reemerging zoonotic viruses (26, 27). IRF1 protein contains a nuclear localization signal (NLS; amino acid residues 117–141) that facilitates the IRF1 nuclear residency (28). The low basal level of IRF1 is present in the nucleus, where it maintains constitutive expression of numerous host defense genes, including *MX1* and *OAS2* (29). Although IRF1 is not essential for the induction of IFN-I genes (30–32), there is clear evidence that IRF1 suppresses the replication of various RNA viruses (33, 34). For example, IRF1 plays a key role in host antiviral defense against Sendai virus (SeV; a paramyxovirus sensed mainly by RIG-I) and DENV (a flavivirus sensed by both RIG-I and MDA5) (33, 35, 36). IRF1-mediated restriction of hepatitis A virus (HAV) replication is independent of MAVS, IRF3, or STAT1 (29). Moreover, IRF1 facilitates TLR9-dependent IFN-β production in mouse dendritic cells (DCs) by interacting with MyD88 (37). Both *IRF1* mRNA and the IRF1 protein are short-lived (38, 39), allowing for rapid and dynamic regulation during viral infection. CHIP, an E3 ligase, degrades IRF-1 by binding to its multifunction domain 2 (Mf2 domain; amino acid residues 106–150) (40). It has also been reported that XAF1 interacts with IRF1, antagonizes CHIP-mediated degradation of IRF1, and eventually inhibits tumorigenesis (19). However, it is unveiled whether XAF1-mediated IRF1 stabilization also facilitates IRF1-dependent host defense against RNA viruses.

In this study, we have found that IRF1 induced XAF1 expression transcriptionally during RNA virus infection. XAF1 stabilizes the IRF1 protein posttranslationally by antagonizing the CHIP-mediated degradation, and thus forms an IRF1-XAF1 positive feedback loop to induce more antiviral IRF1 target genes, including *DDX58*, *DDX60*, *MX1*, and *OAS2*. Our study has described a protective role of XAF1 in the host antiviral innate immunity against RNA viruses.

## RESULTS

### Upregulation of XAF1 expression in hosts infected with RNA viruses.

**FIG 1:**
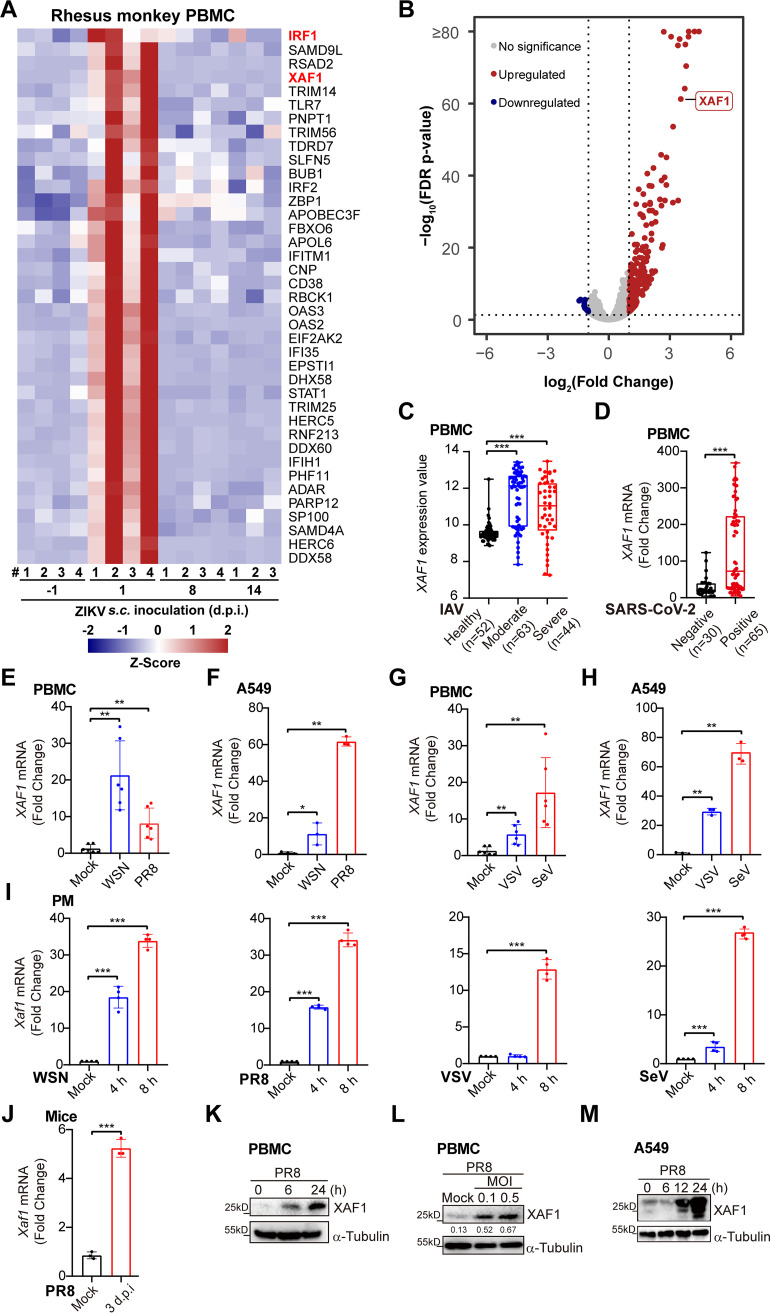
Upregulation of XAF1 expression in host infected with RNA viruses. (A) Heatmap of ISGs in the PBMCs from rhesus monkeys (*n* = 4) subcutaneously injected with ZIKV (10^5^ PFU) for the indicated time points. (B) The volcano plot of differentially expressed ISGs in PBMCs of (A), fold change ≥2 or ≤0.5 (1 d postinfection versus Uninfected control). (C) The PBMCs were from the healthy controls (*n* = 52), moderate influenza patients (*n* = 63), and severe influenza patients (*n* = 44). *XAF1* mRNA expression of these samples was analyzed by microarray. The microarray raw data were downloaded from GEO (accession no. GSE101702). Data are shown as mean ± SD. (D) The PBMCs were from healthy controls (*n* = 30) and SARS-CoV-2-positive COVID-19 patients (*n* = 65). *XAF1* gene expression of these samples was analyzed by RNA-seq. The RNA-seq raw data were downloaded from GEO (accession no. GSE167000). Data are shown as mean ± SD. (E and F) qRT-PCR analysis of *XAF1* mRNA expression in the PBMCs (E) or A549 cells (F) infected with WSN (MOI 0.1) or PR8 (MOI 0.1) for 8 h. (G and H) qRT-PCR analysis of *XAF1* mRNA expression in the PBMCs (G) or A549 cells (H) infected with VSV (MOI 1) or SeV (MOI 0.1) for 8 h. (I) qRT-PCR analysis of *Xaf1* mRNA expression in the PMs infected with WSN (MOI 0.1), PR8 (MOI 0.1), VSV (MOI 1) or SeV (MOI 0.1) for the indicated time points. (J) qRT-PCR analysis of *Xaf1* mRNA expression in the lung tissues from the mice infected with PR8 (100 PFU) intranasally for 3 d. (K and L) Immunoblot analysis of XAF1 in the PBMCs infected with PR8 for the indicated time points (K) or indicated MOI (L). (M) Immunoblot analysis of XAF1 in the A549 cells infected with PR8 (MOI 0.5) for the indicated time points. (E–J) Data from three independent experiments are presented as mean ± SD; *, *P* < 0.05; **, *P* < 0.01; and ***, *P* < 0.001 indicate significant difference by unpaired Student's *t* test. (K–M) Data are representative results of three independent experiments. The band intensities compared to respective loading control were labeled (L).

Infectious diseases caused by the RNA viruses, including influenza A virus (IAV), ZIKV, and SARS-CoV-2, have frequently outbroken in the past several years. To discover the antiviral genes that broadly protect the host against these emerging or reemerging viruses, we sequenced, collected, and reanalyzed multiple transcriptome data sets of immune cells infected with RNA viruses. In the peripheral blood mononuclear cells (PBMCs) from ZIKV-infected rhesus monkeys, induction of XAF1 expression was top ranked among all the antiviral ISGs at 1-day postinfection (Fig. 1A and B). Compared to the PBMCs from the healthy controls, higher expression of XAF1 mRNA was detected in the PBMCs from the moderate and severe influenza patients (Fig. 1C). Similarly, more XAF1 transcripts were detected in the PBMCs from the SARS-CoV-2-positive COVID-19 patients than the healthy controls (Fig. 1D).

Next, we performed viral infection *in vitro* and *in vivo* assays to confirm these phenotypes. Infection with IAV H1N1 strains WSN and PR8 dramatically induced XAF1 transcription in human PBMCs and A549 cells (Fig. 1E and F). Infection with another two RNA viruses, vesicular stomatitis virus (VSV) and SeV, also highly induced XAF1 expression in these human cells (Fig. 1G and H). In addition, upregulation of *XAF1* mRNA expression was observed in the mouse primary peritoneal macrophages (PMs) infected with WSN, PR8, VSV, and SeV in a time-dependent manner (Fig. 1I). We further challenged mice with PR8 intranasally and observed that the *Xaf1* mRNA level was upregulated in the lung tissues of the PR8-infected mice (Fig. 1J). XAF1 protein levels were also induced in the PR8-infected PBMCs and A549 cells in dose-dependent and time-dependent manners (Fig. 1K–M).

Taken together, these results have shown that infection with various RNA viruses, including VSV, SeV, ZIKV, and the life-threatening IAV and SARS-CoV-2, significantly induces XAF1 expression at both mRNA and protein levels *in vitro* and *in vivo*, which suggests the potential role of XAF1 in the regulation of host antiviral immunity against these RNA viruses.

### IRF1 is required for the induction of XAF1 during host antiviral immunity.

**FIG 2:**
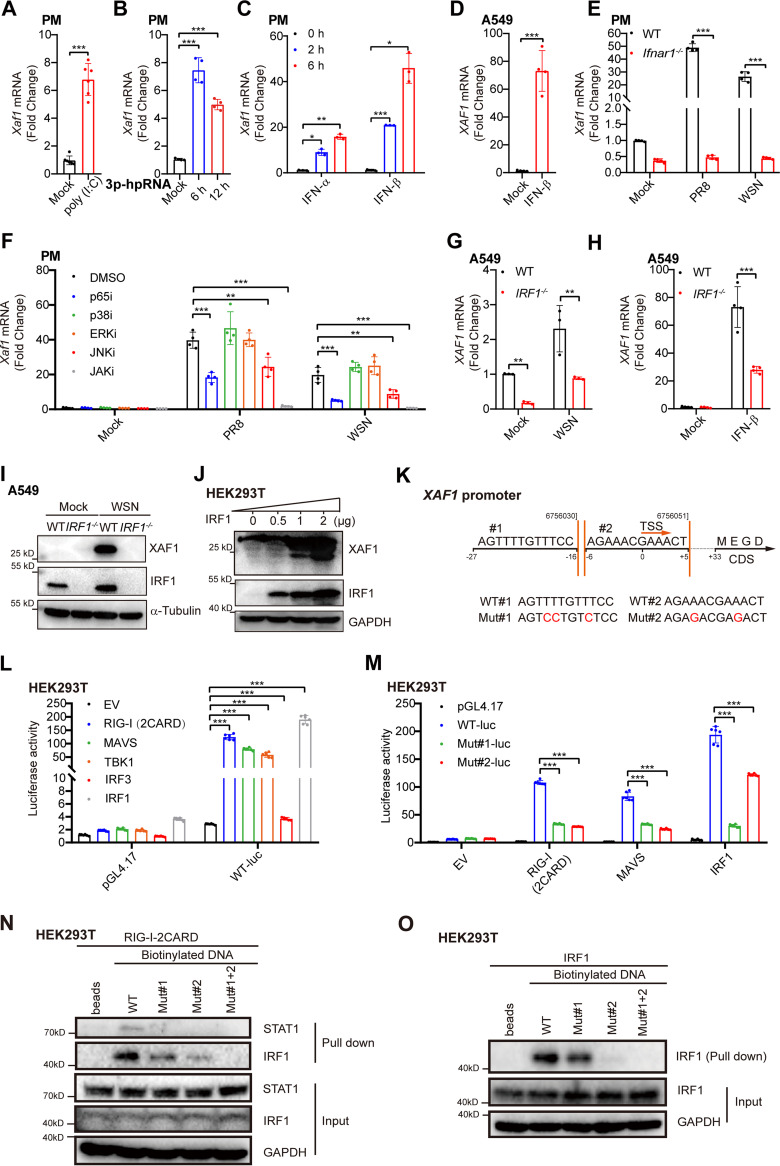
IRF1 is required for the induction of XAF1 during host antiviral immunity. (A) qRT-PCR analysis of *Xaf1* mRNA expression in the PMs transfected with poly(I:C) (1 μg/mL) for 12 h. (B) qRT-PCR analysis of *Xaf1* mRNA expression in the PMs transfected with 3p-hpRNA (200 ng/mL) for the indicated time points. (C) qRT-PCR analysis of *Xaf1* mRNA expression in the PMs treated with IFN-α (500 U/mL) or IFN-β (500 U/mL) for the indicated time points. (D) qRT-PCR analysis of *XAF1* mRNA expression in the A549 cells treated with IFN-β (100 U/mL) for 6 h. (E) qRT-PCR analysis of *Xaf1* mRNA expression in the PMs from the WT and *Ifnar1*^−/−^ mice infected with IAV (PR8 or WSN, MOI 0.1) for 8 h. (F) qRT-PCR analysis of *Xaf1* mRNA expression in the PMs pretreated for 1 h with the indicated inhibitors, followed by IAV infection (PR8 or WSN, MOI 0.1) for 8 h. (G) qRT-PCR analysis of *XAF1* mRNA expression in the WT and *IRF1*^−/−^ A549 cells infected with WSN (MOI 0.1) for 8 h. (H) qRT-PCR analysis of *XAF1* mRNA expression in the WT and *IRF1*^−/−^ A549 cells stimulated with IFN-β (100 U/mL) for 8 h. (I) Immunoblot analysis of indicated proteins in the WT and *IRF1*^−/−^ A549 cells infected with WSN (MOI 0.1) for 12 h. (J) The IRF1 plasmid (0, 0.5, 1, 2 μg) were transfected into HEK293T cells in a 6-well plate. After 24 h, total cell lysates were subjected to immunoblot analysis of indicated proteins. (K) The model of potential IRF1 binding sites in XAF1 promoter. (L) EV (empty vector), RIG-I(2CARD), MAVS, TBK1, IRF3, or IRF1 vector (100 ng) was cotransfected with the control vector pGL4.17 (100 ng), XAF1 promoter-luciferase reporter constructs (WT-luc, 100 ng), and pRL-TK-Luc vector (10 ng) into HEK293T cells in a 24-well plate. After 24 h, relative luciferase activity was quantified. (M) EV, RIG (2CARD), MAVS, or IRF1 vector (100 ng) was cotransfected with the control vector pGL4.17 (100 ng), XAF1 promoter-luciferase reporter constructs (WT-luc, 100 ng), or mutant XAF1 promoter-luciferase reporter constructs (Mut-luc, 100 ng), and pRL-TK-Luc vector (10 ng) into HEK293T cells in a 24-well plate. After 24 h, relative luciferase activity was quantified. (N) The RIG-I(2CARD) plasmid (5 μg) was transfected into HEK293T cells in a 10-cm dish. After 24 h, total cell lysates were incubated with biotinylated DNA (Mut#1, Mut#2, Mut#1 + 2 [double mutation], respectively), followed by immunoblot detection. (O) The IRF1 plasmid (5 μg) was transfected into HEK293T cells in a 10-cm dish. After 24 h, total cell lysates were incubated with biotinylated DNA (Mut#1, Mut#2, Mut#1 + 2, respectively), followed by immunoblot detection. (A–H, L, and M) Data from three independent experiments are presented as mean ± SD; *, *P < *0.05; **, *P < *0.01; and ***, *P < *0.001 indicate significant difference by unpaired Student's *t* test. (I, J, N, and O) Data are representative results of three independent experiments.

To explore the signaling pathways that control the induction of XAF1 during viral infection, we transfected PMs with two viral RNA mimics, poly(I:C) and 3p-hpRNA, which potently activate the RIG-I-MAVS-TBK1-IRF3-IFN-I signaling axis (41, 42). Both RNA mimics significantly induced XAF1 transcription (Fig. 2A and B), which suggested that viral RNA-triggered activation of the RIG-I signaling pathway was essential for the induction of XAF1. IFN-I, including IFN-α and IFN-β, two key products of activated RIG-I signaling, induced XAF1 expression in PMs and A549 cells (Fig. 2C and D). During PR8 and WSN infection, much less induction of XAF1 was observed in the *Ifnar1^−/−^* PMs than in the wide-type (WT) cells (Fig. 2E and Fig. S1A). Activation of NF-κB, JAK-STAT1/2, and MAPK signaling pathways is required to fully induce IFN-I, ISGs, and proinflammatory cytokines during antiviral immunity (10, 43). Specific inhibitors targeting NF-κB p65, MAPK JNK, and JAK significantly attenuated the induction of XAF1 during the PMs infected with PR8 and WSN (Fig. 2F and Fig. S1B–F). These results indicated that activating NF-κB, JNK, and JAK was required to induce XAF1 during RNA virus infection.

Next, we analyzed the underlying transcription factors that controlled XAF1 expression. It has been reported that induction of IRF1 expression is under the control of NF-κB (39), JNK (44), and JAK-STAT1 (15). XAF1 transcription is activated by IRF1 under stressful conditions (19). Therefore, we speculated that induction of the transcription factor IRF1 determined the elevated XAF1 expression during viral infection. Infection with RNA viruses, including WSN, PR8, VSV, and SeV induced IRF1 expression (Fig. S2A). IRF1 bound to the promoter region of *Xaf1*, and treatment with IFN-γ, a potent JAK-STAT1 activator, enhanced this binding (Fig. S2B). Knockout of IRF1 significantly reduced the induction of *XAF1* transcription during WSN infection and IFN-β treatment (Fig. 2G and H, and Fig. S2C). Almost no XAF1 proteins were detected in the WSN-infected *IRF1^−/−^* A549 cells (Fig. 2I). However, overexpression of IRF1 gradually induced XAF1 protein in a dose-dependent manner in HEK293T cells (Fig. 2J). Two potential IRF-binding elements (IRF-Es) were predicted in the promoter of *XAF1* (Fig. 2K). IRF1 and the key adaptors in the RIG-I signaling pathways dramatically induced the XAF1 transcriptional activity (Fig. 2L). However, mutation of IRF-E1 or IRF-E2 significantly abolished the transcriptional activity of *XAF1* induced by the overexpressed RIG-I, MAVS, and IRF1 (Fig. 2M). Biotinylated DNA that contained the two IRF-Es of the *XAF1* promoter interacted with IRF1 rather than STAT1 in the RIG-I-overexpressed HEK293T cells (Fig. 2N), although the IRF-E and IFN-sensitive response element (ISRE) have very similar sequences. In RIG-I or IRF1-overexpressed HEK293T cells, the core region of the XAF1 promoter with mutant IRF-E1, IRF-E2, or both inhibited biotinylated DNA-IRF1 binding (Fig. 2N and O). These results indicated that the transcription factor IRF1 bound to the IRF-Es in the *XAF1* promoter and induced XAF1 transcription during host antiviral immunity against RNA viruses.

### XAF1 protects the host against multiple RNA viruses.

**FIG 3:**
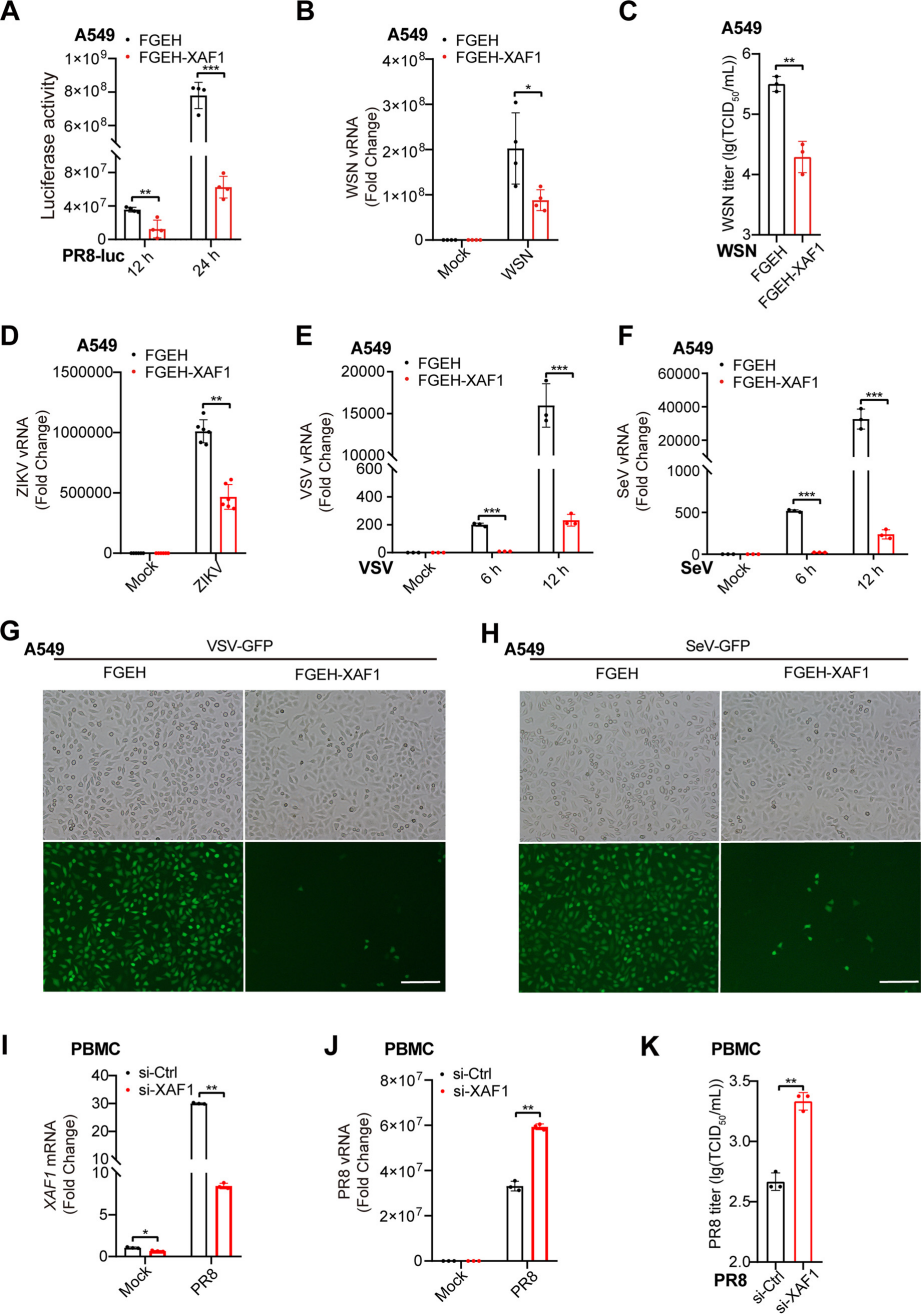
XAF1 protects the host against multiple RNA viruses. (A) The FGEH and FGEH-XAF1 A549 cell clones were infected with PR8-Luc (MOI 1). After 12 h or 24 h, relative renilla luciferase activity was quantified. (B) qRT-PCR analysis of viral RNA (vRNA) in the FGEH and FGEH-XAF1 A549 cell clones infected with WSN (MOI 0.1) for 8 h. (C) Supernatants of (B) were measured for WSN TCID_50_ assays. (D) qRT-PCR analysis of viral RNA in the FGEH and FGEH-XAF1 A549 cell clones infected with ZIKV (MOI 0.01) for 24 h. (E and F) qRT-PCR analysis of viral RNA in the FGEH and FGEH-XAF1 A549 cell clones infected with VSV (MOI 1) (E), and SeV (MOI 0.1) (F) for the indicated time points. (G and H) Fluorescence microscopy imaging of GFP in the FGEH and FGEH-XAF1 A549 cell clones infected with VSV-GFP (MOI 1) (G), and SeV-GFP (MOI 0.1) (H) for 8 h; scale bar, 200 μm. (I) qRT-PCR analysis of *XAF1* mRNA in the PBMCs transfected with control (Ctrl) or XAF1 siRNA for 48 h, followed by PR8 infection (MOI 0.1) for 12 h. (J) qRT-PCR analysis of viral RNA in the PBMCs transfected with Ctrl or XAF1 siRNA for 48 h, followed by PR8 infection (MOI 0.1) for 12 h. (K) Supernatants of (J) were measured for PR8 TCID_50_ assays. (A–F and I–K) Data from three independent experiments are presented as mean ± SD; *, *P < *0.05; **, *P < *0.01; and ***, *P < *0.001 indicate significant difference by unpaired Student's *t* test. Data of (G and H) are representative results of three independent experiments.

To investigate the function of XAF1 in host antiviral immunity, we stably overexpressed XAF1 in A549 cells via a lentiviral gene delivery system (Fig. S3A). Overexpression of XAF1 inhibited the luciferase activity in PR8-Luc-infected A549 cells (Fig. 3A). In the WSN-infected A549 cells, less viral RNA and supernatant viral particles were detected in the XAF1-overexpressed cells than in the control cells (Fig. 3B and C). Consistently, overexpression of XAF1 inhibited the viral RNA expression in the ZIKV-, VSV-, or SeV-infected A549 cells (Fig. 3D–F). Much fewer green fluorescent protein (GFP)-positive cells and less GFP fluorescence were observed in the VSV-GFP-infected or SeV-GFP-infected A549 cells than in the control cells (Fig. 3G and H and Fig. S3B, C). These results indicate that overexpression of XAF1 broadly inhibits the replication of RNA viruses, including IAV, ZIKV, VSV, and SeV. In addition, more viral RNA and supernatant viral particles were detected in the PBMCs where the XAF1 was knocked down via RNA interference (RNAi) (Fig. 3I–K), which suggested a protective role of XAF1 in human primary immune cells. Taken together, these results demonstrated that upregulation of XAF1 expression during viral infection broadly protected the host against RNA viruses.

### Knockout of XAF1 facilitates RNA virus infection *in vitro* and *in vivo*.

**FIG 4:**
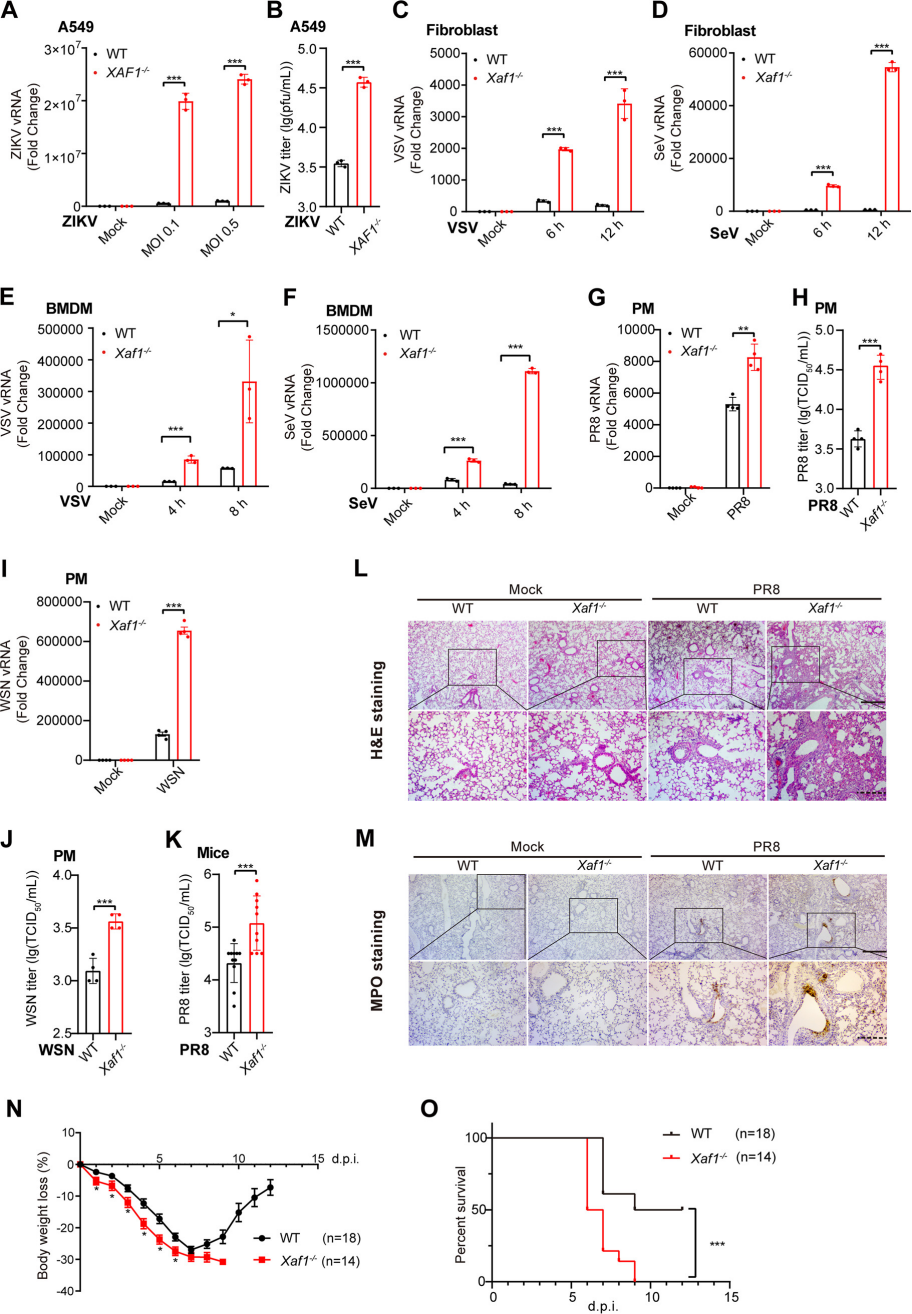
Knockout of XAF1 facilitates RNA virus infection *in vitro* and *in vivo.* (A) qRT-PCR analysis of viral RNA in the WT and *XAF1*^−/−^ A549 cell clones infected with ZIKV for 24 h. (B) Supernatants of (A) were measured for ZIKV plaque assays. (C and D) qRT-PCR analysis of viral RNA in the WT and *Xaf1*^−/−^ fibroblast infected with VSV (MOI 1) (C) or SeV (MOI 0.1) (D) for the indicated time points. (E and F) qRT-PCR analysis of viral RNA in the WT and *Xaf1*^−/−^ BMDMs infected with VSV (MOI 1) (E) or SeV (MOI 0.1) (F) for the indicated time points. (G) qRT-PCR analysis of viral RNA in the WT and *Xaf1*^−/−^ PMs infected with PR8 (MOI 0.1) for 8 h. (H) TCID_50_ assays of supernatants from the WT and *Xaf1*^−/−^ PMs infected with PR8 (MOI 0.1) for 12 h. (I) qRT-PCR analysis of viral RNA in the WT and *Xaf1*^−/−^ PMs infected with WSN (MOI 0.1) for 8 h. (J) TCID_50_ assays of supernatants from the WT and *Xaf1*^−/−^ PMs infected with WSN (MOI 0.1) for 12 h. (K) TCID_50_ assays of the lung homogenates from the WT (*n* = 11) and *Xaf1*^−/−^ (*n* = 10) mice infected with PR8 (100 PFU) intranasally for 5 d. (L and M) Histological examination of the lung sections from the WT and *Xaf1*^−/−^ mice infected with PR8 (100 PFU) intranasally for 5 d was performed by H&E staining (L), and neutrophil infiltrations of the lung sections from the indicated mice were measured by MPO staining (M); scale bar, “—” represents 400 μm; “‐‐‐” represents 200 μm; data are representative results of three independent experiments. (N and O) Survival assays of the WT and *Xaf1*^−/−^ mice infected with PR8 (100 PFU) intranasally (WT group: *n* = 18; *Xaf1*^−/−^ group: *n* = 14). Bodyweight loss curves (N) and Kaplan-Meier survival curves (O) were generated and analyzed from three independent experiments; *, *P < *0.05 and ***, *P < *0.001 indicate significant difference. Data of (A–K) from three independent experiments are presented as mean ± SD; *, *P < *0.05; **, *P < *0.01; and ***, *P < *0.001 indicate significant difference by unpaired Student's *t* test.

We further validated the physiological function of XAF1 during host fighting against the invaded RNA viruses. In A549 cells, much more ZIKV RNA was detected in the *XAF1^−/−^* cells than in the WT cells (Fig. 4A). Plaque assay results also indicated that more viral particles were released into the supernatant of *XAF1^−/−^* cells than the WT cells (Fig. 4B). Next, we deleted the *Xaf1* in mice by CRISPR/Cas9 technology and confirmed the knockout efficiency in mouse organs and PMs by PCR or RT-qPCR (Fig. S4A–D). More viral RNA was detected in the *Xaf1^−/−^* fibroblast and bone marrow-derived macrophages (BMDMs) than in the WT cells during VSV and SeV infection (Fig. 4C–F). During PR8 and WSN infection, more viral RNA in the *Xaf1^−/−^* PMs and more viral particles in the *Xaf1*^−/−^ PMs supernatant were detected than in their respective WT controls (Fig. 4G–J). In addition to these results from the *ex vivo* experiments, more viruses were detected in the lung homogenate from the *Xaf1*^−/−^ mice than the WT group in the mice infected with PR8 intranasally (Fig. 4K). More severe lung injuries, including alveolar wall thickening, alveolar cavity atrophy, and neutrophil infiltration, were observed in the PR8-infected *Xaf1^−/−^* mice than in the WT mice (Fig. 4L and M and Fig. S4E). Similarly, VSV infection intraperitoneally led to much more severe lung injuries in the *Xaf1^−/−^* mice than in the WT mice (Fig. S4F). Next, WT and *Xaf1*^−/−^ mice were challenged with a lethal dose of PR8 intranasally, and the bodyweight loss and survival rate for 12 days were checked. *Xaf1*^−/−^ mice were more susceptible to PR8-induced body-weight loss and lethality than the WT mice (Fig. 4N and O).

In summary, *in vitro*, *ex vivo*, and *in vivo* experiments consistently indicated that knockout of XAF1 facilitated RNA virus infection. XAF1 was essential in the host fighting against numerous RNA viruses, including the life-threatening IAV.

### Attenuated induction of IRF1 target genes in the *Xaf1^–/–^* macrophages.

We sought to explore the underlying molecular mechanism for the protective role of XAF1 during viral infection. First, given that XAF1 is an ISG that enhances IFN-induced apoptosis (14, 15), we checked the proapoptotic function of XAF1 during IAV infection. PR8 infection significantly induced apoptosis of A549 cells. However, overexpression of XAF1 did not affect the percentage of the PR8-induced apoptosis (Fig. S5A). In the VSV- and SeV-infected BMDMs, knockout of *Xaf1* significantly reduced the activation of RIG-I-like receptors and defense response to virus signaling pathways. However, there was no significant difference in the apoptosis signaling pathway between WT and *Xaf1^−/−^* cells during viral infection (Fig. S5B and C).

**FIG 5:**
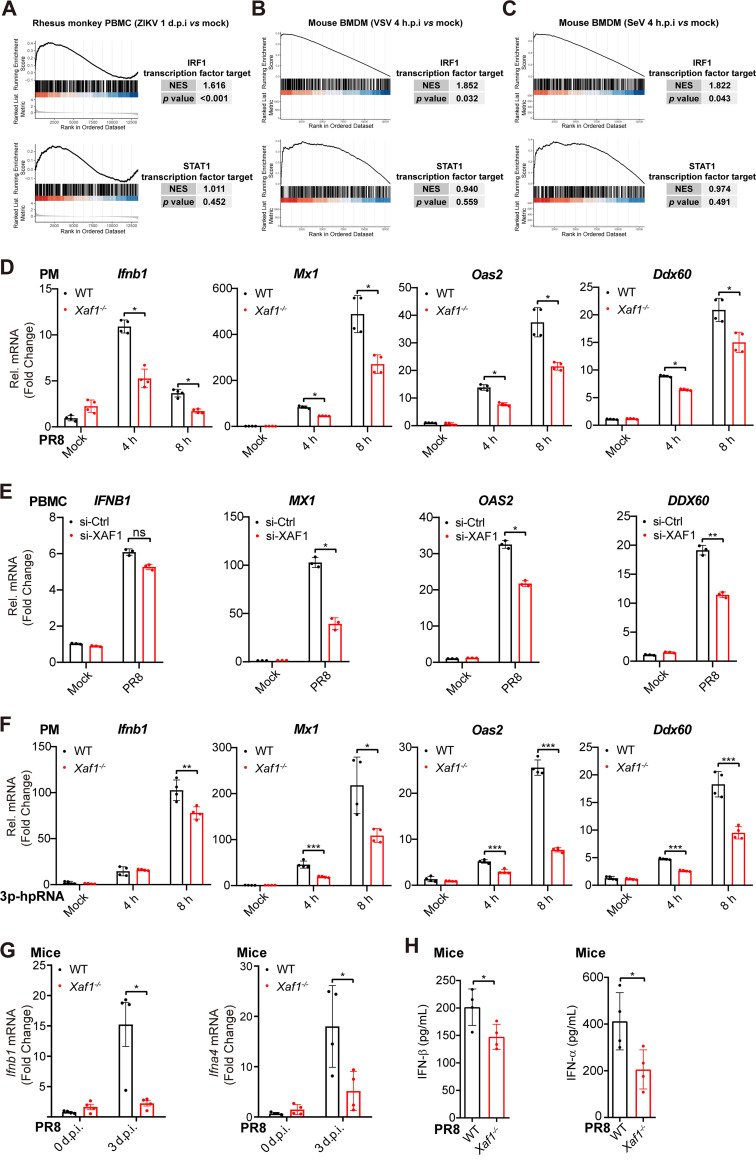
Attenuated induction of IRF1 target genes in the *Xaf1*^−/−^ macrophages. (A–C) GSEA analysis of IRF1 and STAT1 transcription factor targeted gene sets for ZIKV-infected rhesus monkey PBMCs (A), VSV-infected mouse BMDMs (B), and SeV-infected mouse BMDMs (C), compared with their uninfected status. normalized enrichment scores (NES) > 1 and *P* value < 0.05 indicate significantly upregulated expression of IRF1 target genes after infection than STAT1 target genes. (D) qRT-PCR analysis of *Ifnb1* and antiviral ISGs (*Mx1*, *Oas2*, and *Ddx60*) mRNA levels in the WT and *Xaf1*^−/−^ PMs infected with PR8 (MOI 0.1) for the indicated time points. (E) qRT-PCR analysis of *IFNB1* and antiviral ISGs (*MX1*, *OAS2*, and *DDX60*) mRNA levels in the PBMCs transfected with Ctrl or XAF1 siRNA for 48 h, followed by PR8 infection (MOI 0.1) for 12 h. (F) qRT-PCR analysis of *Ifnb1* and antiviral ISGs (*Mx1*, *Oas2*, *Ddx60*) mRNA levels in the WT and *Xaf1*^−/−^ PMs transfected with 3p-hpRNA (200 ng/mL) for the indicated time points. (G) qRT-PCR analysis of *Ifnb1* and *Ifna4* mRNA levels in lung tissues from the WT and *Xaf1*^−/−^ mice (*n* = 4, respectively) infected with PR8 (100 PFU) intranasally for 3 d. (H) ELISA of IFN-β and IFN-α in lung homogenate from the WT and *Xaf1*^−/−^ mice (*n* = 4, respectively) infected with PR8 (100 PFU) intranasally for 5 d. (D–H) Data from three independent experiments are presented as mean ± SD; ns, no significance; *, *P < *0.05; **, *P < *0.01; and ***, *P < *0.001 indicate significant difference by unpaired Student's *t* test.

Among all the differentially expressed genes in the immune cells infected with RNA viruses (e.g., ZIKV, VSV, and SeV), IRF1 target genes were highly and significantly enriched (Fig. 5A–C). Combined with previous research and the list of IRF1 target genes in the Molecular Signatures Database (Table S1), we selected several antiviral genes as the representative IRF1 target genes, including *DDX58*, *MX1*, *OAS2*, *and DDX60* (45). The transcripts of these genes were all highly induced during viral infection and contain IRF-E motifs in their promoter regions (Fig. S5D–F). Less induction of the *Ifnb1*, *Mx1*, *Oas2*, and *Ddx60* transcripts was observed in the PR8-infected *Xaf1^−/−^* PMs than in the WT cells (Fig. 5D). Knockdown of *XAF1* in human PBMCs attenuated the PR8 infection-triggered induction of the *MX1*, *OAS2*, and *DDX60* transcripts, but not the *IFNB1* transcripts (Fig. 5E). Viral RNA mimic 3p-hpRNA induced more transcripts of the *Ifnb1* and IRF1 target genes, including *Mx1*, *Oas2*, and *Ddx60*, in the WT PMs than in the *Xaf1^−/−^* PMs (Fig. 5F). In addition, fewer *Ifnb1* and *Ifna4* transcripts were detected in the lungs of the PR8-infected *Xaf1^−/−^* mice than in the WT mice (Fig. 5G). Less IFN-α and IFN-β were detected in the lung homogenate from the PR8-infected *Xaf1^−/−^* mice than in the WT mice (Fig. 5H).

These results indicated that the induction of IRF1 target genes such as *MX1*, *OAS2*, and *DDX60* was a defect in the RNA virus-infected *XAF1^−/−^* immune cells.

### XAF1 facilitates IRF1-dependent induction of antiviral ISGs.

**FIG 6:**
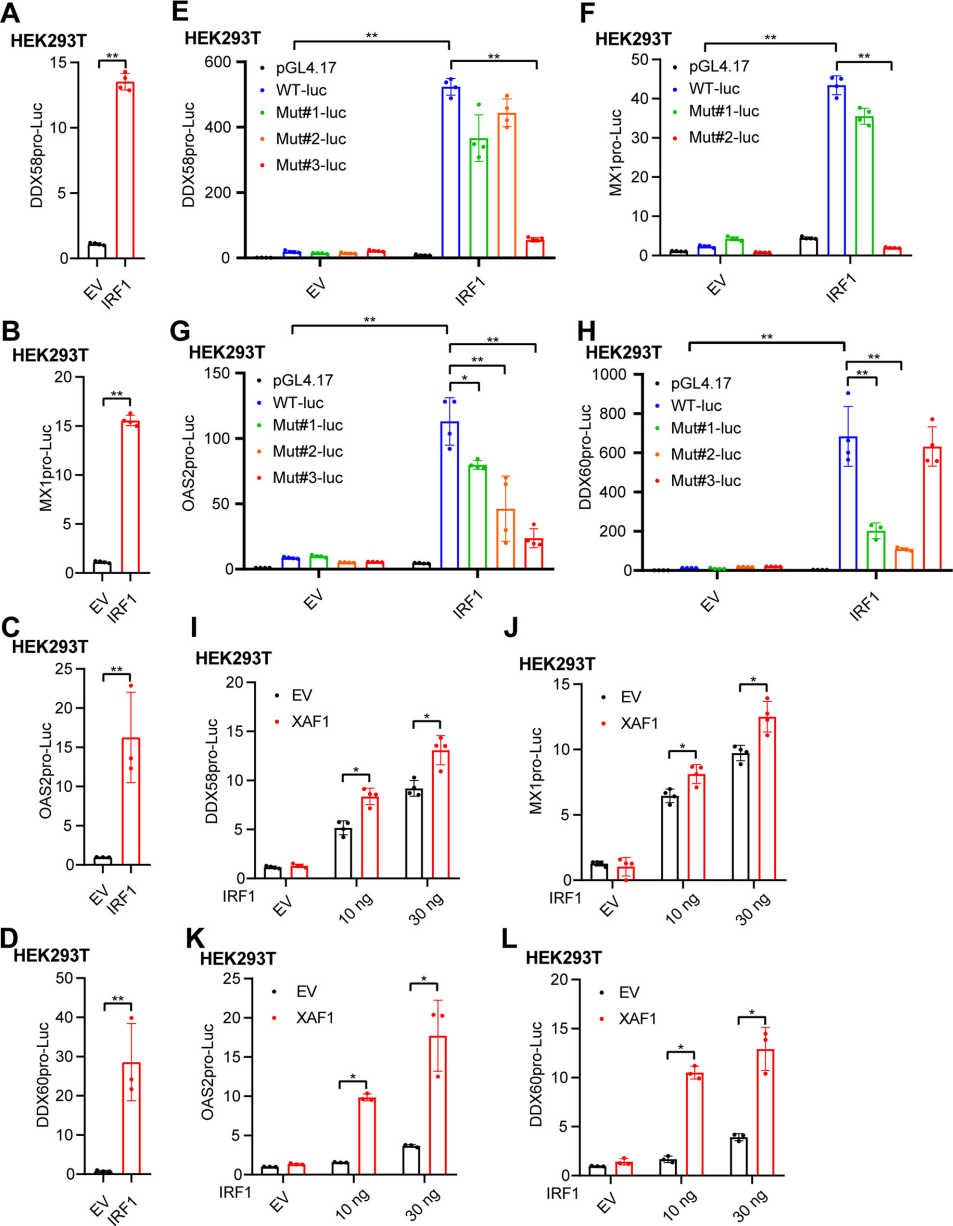
XAF1 facilitates IRF1-dependent induction of antiviral ISGs. (A–D) The IRF1 plasmid (100 ng) was cotransfected with the pRL-TK-Luc vector (10 ng), and *DDX58* (A), *MX1* (B), *OAS2* (C), or *DDX60* (D) promoter-luciferase reporter vector (100 ng each) into HEK293T cells in a 24-well plate. After 24 h, relative luciferase activity was quantified. (E–H) The IRF1 plasmid (100 ng) was cotransfected with the pRL-TK-Luc vector (10 ng) and the control vector (pGL4.17), *DDX58*, *MX1*, *OAS2*, *DDX60* promoter-luciferase reporter vector, or mutant vector (100 ng each) into HEK293T cells in a 24-well plate. After 24 h, DDX58 (E), MX1 (F), OAS2 (G), or DDX60 (H) luciferase activity was quantified. (I–L) The XAF1 (100 ng) and IRF1 (0, 10, and 30 ng) plasmids were cotransfected with the pRL-TK-Luc vector (10 ng), and *DDX58* (I), *MX1* (J), *OAS2* (K), or *DDX60* (L) promoter-luciferase reporter vector (100 ng each) into HEK293T cells in a 24-well plate. After 24 h, relative luciferase activity was quantified. All data from three independent experiments are presented as mean ± SD; *, *P < *0.05 and **, *P < *0.01 indicate significant difference by unpaired Student's *t* test.

IRF1 plays a powerful role in antiviral immunity mainly through its transcriptional activity (29, 46). We tried to investigate how IRF1 regulated the transcription of *DDX58*, *MX1*, *OAS2*, and *DDX60*. The promoters of these genes were cloned into the pGL4.17 promoter reporter vector, and overexpression of IRF1 dramatically induced the transcription activity of these genes (Fig. 6A–D). The IRF-Es in the IRF1 binding region were predicted, mutated, and cloned into the promoter reporter vector (Fig. S5E, F and Fig. S6). Three IRF-Es were located in the promoter of *DDX58*, and the third one was critical for the induction of *DDX58* transcription by IRF1 (Fig. 6E and Fig. S6A). Similarly, the second IRF-E of *MX1*, all the three IRF-Es of *OAS2*, and the first and second IRF-Es of *DDX60* were essential for the transcriptional induction of *MX1*, *OAS2*, and *DDX60*, respectively (Fig. 6F–H and Fig. S6B–D). Overexpression of XAF1 further facilitated the transcription activities of *DDX58*, *MX1*, OAS2, and *DDX60* (Fig. 6I–L). Together, the results from these promoter reporters demonstrated that XAF1 facilitated IRF1-dependent induction of the antiviral ISGs, including DDX58, MX1, OAS2, and DDX60.

### XAF1 maintains IRF1 expression and IRF1-dependent antiviral immunity.

**FIG 7:**
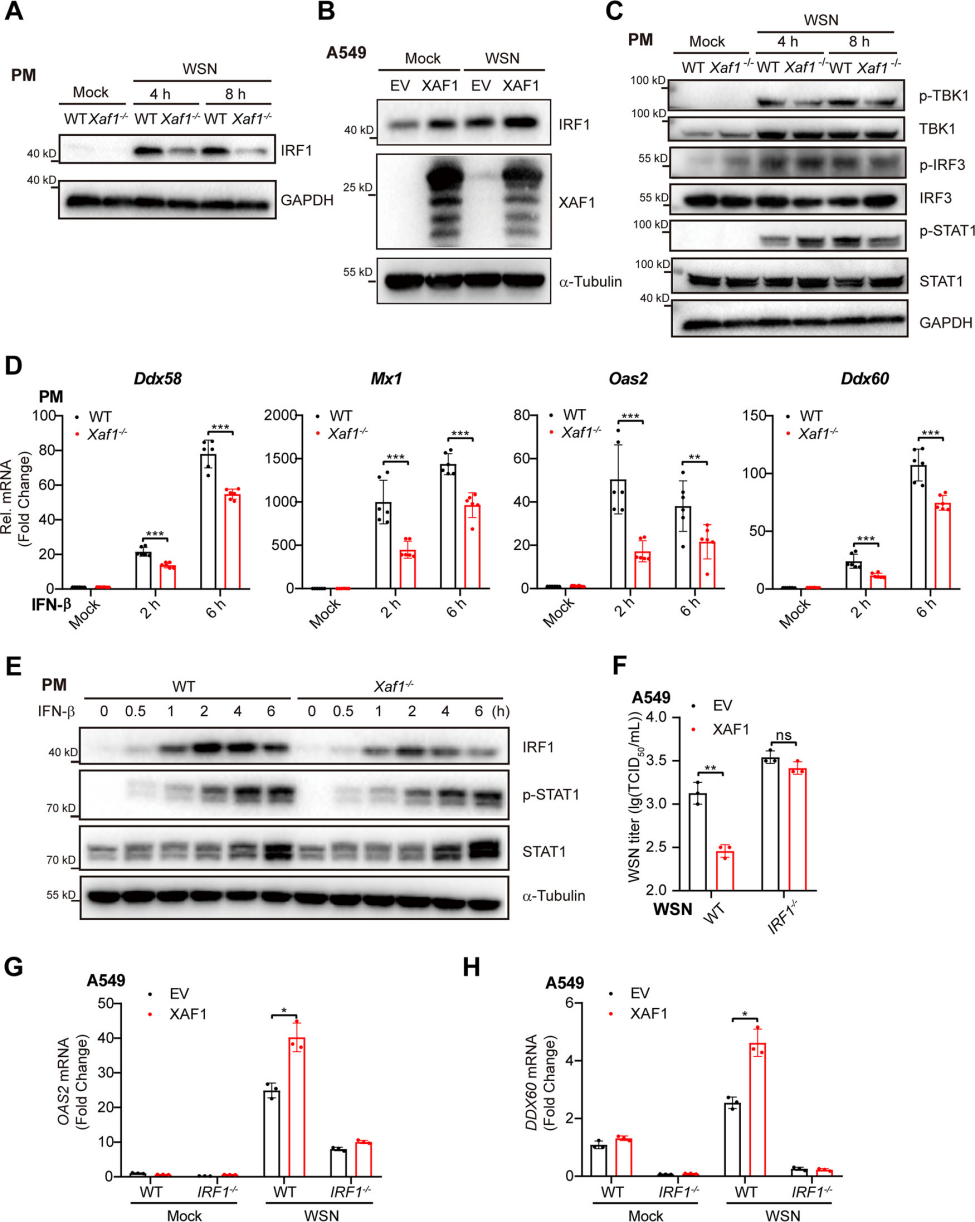
XAF1 maintains IRF1 expression and IRF1-dependent antiviral immunity. (A) Immunoblot analysis of IRF1 protein in the WT and *Xaf1*^−/−^ PMs infected with WSN (MOI 0.1) at the indicated time points. (B) Immunoblot analysis of indicated proteins. A549 cells in a 6-well plate were transfected with the vector or XAF1 (1 μg) plasmid, followed by WSN (MOI 0.1) infection for 6 h. (C) Immunoblot analysis of indicated proteins in the WT and *Xaf1*^−/−^ PMs infected with WSN (MOI 0.1) for the indicated time points. (D) qRT-PCR analysis of antiviral ISGs (*Ddx58*, *Mx1*, *Oas2*, and *Ddx60*) mRNA levels in the WT and *Xaf1*^−/−^ PMs treated with IFN-β (500 U/mL) for the indicated time points. (E) Immunoblot analysis of indicated proteins in the WT and *Xaf1*^−/−^ PMs treated with IFN-β (500 U/mL) at the indicated time points. (F) WT and *IRF1^−/−^* A549 cells in 12-well plates were transfected with the EV or XAF1 (0.5 μg) plasmid, followed by WSN (MOI 0.1) infection for 12 h. The supernatants were measured for TCID_50_ assays. (G and H) The WT and *IRF1^−/−^* A549 cells in 12-well plates were transfected with the empty vector (EV) or XAF1 (0.5 μg) plasmid, followed by WSN (MOI 0.1) infection for 6 h. qRT-PCR analysis of *OAS2* (G) and *DDX60* (H) mRNA levels in cell lysate. (D, F, G, and H) Data from three independent experiments are presented as mean ± SD; *, *P < *0.05; **, *P < *0.01; and ***, *P < *0.001 indicate significant difference, and ns, no significant difference by unpaired Student's *t* test. Data of (A–C and E) are representative results of three independent experiments.

To investigate how XAF1 facilitates the induction of IRF1 target genes, we first measured the IRF1 expression during viral infection. WSN infection induced IRF1 proteins in WT PMs, while attenuated induction of IRF1 was observed in the *Xaf1^−/−^* PMs (Fig. 7A). Overexpression of XAF1 significantly upregulated the IRF1 protein levels in both uninfected and WSN-infected A549 cells (Fig. 7B). Next, we further determined whether XAF1 regulated IFN-I upstream or downstream signaling. In the WSN-infected PMs, more phosphorylation of STAT1 at the early stage (4 h postinfection) but less phosphorylation of STAT1 at the later stage (8 h postinfection) was detected in the *Xaf1^−/−^* PMs than in the WT cells. However, the phosphorylation of TBK1 and IRF3, the IFN-I upstream signaling, was similar in the WSN-infected WT and *Xaf1^−/−^* PMs (Fig. 7C). These results suggested that XAF1 mainly regulated the IFN-I downstream signaling.

IFN-β induced the IRF1 target genes, including *DDX58*, *MX1*, *OAS2*, and *DDX60* in HEK293T cells, while knockdown of IRF1 via RNAi attenuated the induction of these genes by IFN-β (Fig. S7). Induction of these IRF1 target genes was impaired in the IFN-β-treated *Xaf1^−/−^* PMs than the WT cells (Fig. 7D), which drove us to check whether the induction of IRF1 by IFN-β was also attenuated in *Xaf1^−/−^* PMs. IFN-β robustly induced IRF1 protein as early as 30 min posttreatment. However, much less induction of IRF1 was observed in the *Xaf1^−/−^* PMs than in the WT cells (Fig. 7E). In the IFN-β-stimulated PMs, phosphorylation of STAT1 was later than the induction of IRF1 protein, and there was no difference in phosphorylated STAT1 between WT and *Xaf1^−/−^* cells (Fig. 7E). Overexpression of XAF1 inhibits WSN infection in the WT A549 cells but not in the *IRF1^−/−^* cells (Fig. 7F). Consistently, overexpression of XAF1 facilitated the induction of the OAS2 and DDX60 in the WT A549 cells but not in the *IRF1^−/−^* cells (Fig. 7G and H).

Together, these results indicated that XAF1 maintained the IRF1 expression and IRF1-dependent antiviral immunity. IRF1 mediated the protective effect of XAF1 during host cells fighting against RNA viruses.

### XAF1 inhibits the proteasome degradation of IRF1.

**FIG 8:**
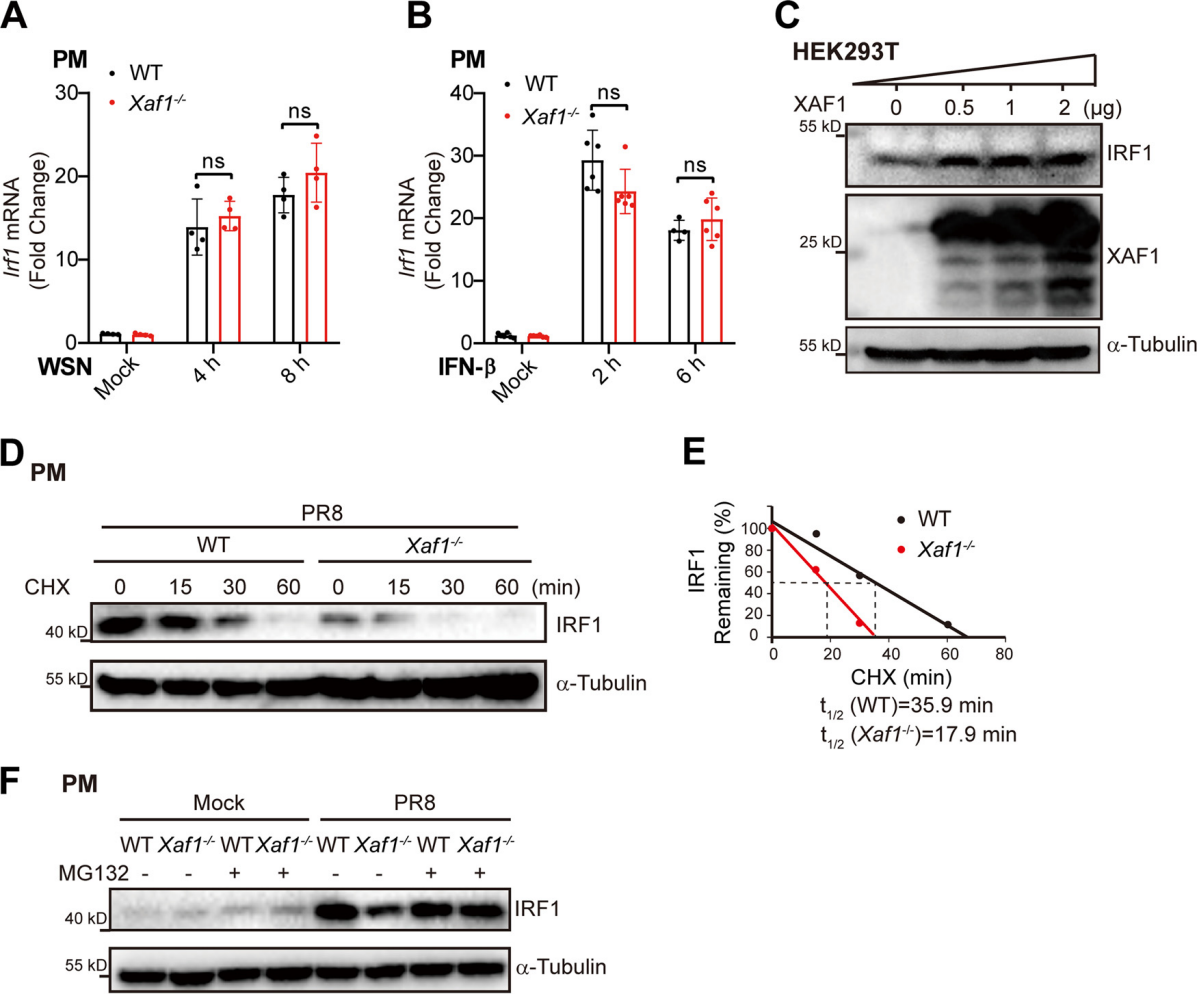
XAF1 inhibits the proteasome degradation of IRF1. (A) qRT-PCR analysis of *Irf1* mRNA level in the WT and *Xaf1*^−/−^ PMs infected with WSN (MOI 0.1) for the indicated time points. (B) qRT-PCR analysis of *Irf1* mRNA level in the WT and *Xaf1*^−/−^ PMs treated with IFN-β (500 U/mL) for the indicated time points. (C) The XAF1 plasmid (0, 0.5, 1, or 2 μg) was transfected into HEK293T cells in a 6-well plate. After 24 h, total cell lysates were subjected to immunoblot analysis of indicated proteins. (D and E) The WT and *Xaf1*^−/−^ PMs were infected with PR8 (MOI 0.1) for 8 h. After CHX (100 μg/mL) treatment, cells were harvested at the indicated time points, and cell lysates were subjected to immunoblot analysis (D); and the optical density of IRF1 protein bands was analyzed with ImageJ software (E). (F) The WT and *Xaf1*^−/−^ PMs were infected with PR8 (MOI 0.1) for 6 h, followed by MG132 (10 μM) treatment for 6 h. Cell lysates were subjected to immunoblot analysis. (A and B) Data from three independent experiments are presented as mean ± SD; ns, no significant difference by unpaired Student's *t* test. (C–F) Data are representative results of three independent experiments.

To clarify how XAF1 upregulates IRF1 expression, we compared the IRF1 mRNA level in the WT and *Xaf1*^−/−^ macrophages infected with WSN or treated with IFN-β. There was no difference in IRF1 mRNA level between WT and *Xaf1*^−/−^ macrophages (Fig. 8A and B), which indicated that XAF1 did not affect IRF1 transcription during viral infection. Overexpression of XAF1 upregulated IRF1 protein levels in a dose-dependent manner in the HEK293T cells without any infection or stimulation (Fig. 8C), which further suggested that XAF1 regulates the turnover of the IRF1 protein. Next, we checked the half-life of the IRF1 protein in the PR8-infected PMs by the cycloheximide (CHX) chase assay. Much less IRF1 was left in the *Xaf1^−/−^* PMs compared to WT cells. The half-life of the endogenous IRF1 protein induced by PR8 infection was shortened more in the *Xaf1*^−/−^ PMs than in the WT cells (Fig. 8D and E). These results suggested that XAF1 potentially inhibits IRF1 protein degradation. The proteasome pathway commonly contributes to protein degradation in eukaryotic cells. Blocking proteasome-mediated protein degradation by the inhibitor MG132 completely restored the IRF1 protein level in the PR8-infected *Xaf1^−/−^* PMs (Fig. 8F), indicating that XAF1 stabilizes the IRF1 protein by suppressing the proteasome pathway.

### XAF1 stabilizes IRF1 protein by antagonizing CHIP-mediated degradation.

**FIG 9:**
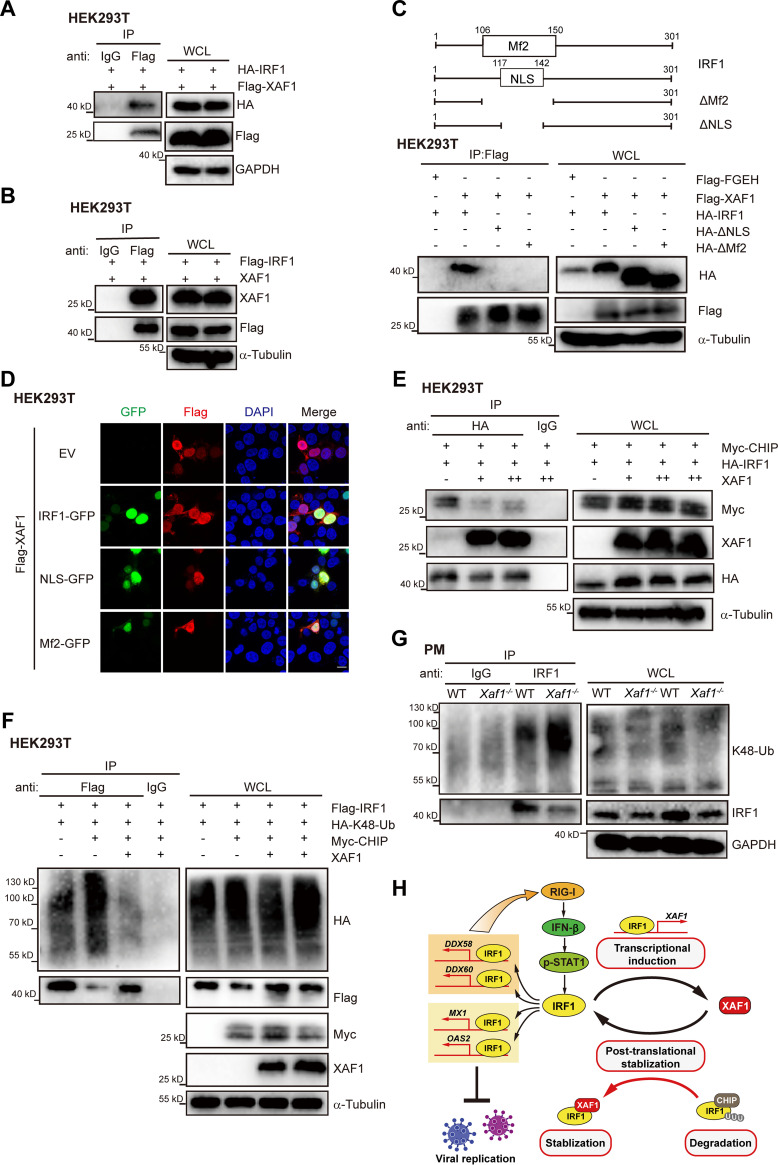
XAF1 stabilizes IRF1 protein by antagonizing CHIP-mediated degradation. (A and B) Immunoprecipitation analysis of the interaction of exogenous XAF1-IRF1 in HEK293T cells in a 10-cm dish transfected with the XAF1 (4 μg) and IRF1 (4 μg) plasmids for 24 h. (C) WT and truncated mutants of IRF1 (2 μg) were cotransfected with Flag-Flag-FGEH (2 μg) or Flag-XAF1 (2 μg) into HEK293T cells seeded in 60-mm dishes for 24 h, and anti-FLAG immunoprecipitates from total cell lysates were subjected to Immunoblot (IB) analysis. (D) Immunofluorescence microscopic colocalization analysis of XAF1 and IRF1. HEK293T cells transfected with the Flag-XAF1 (1 μg) and EV, IRF1-GFP, NLS-GFP, or Mf2-GFP (1 μg) for 24 h were incubated with anti-Flag antibody, and proteins were visualized with secondary antibodies; scale bar, “—” represents 25 μm. (E) Immunoprecipitation analysis of the interaction of exogenous IRF1-CHIP in HEK293T cells in a 10-cm dish transfected with the indicated plasmids for 24 h. (F) The ubiquitination of exogenous IRF1 was analyzed by IP and immunoblot in HEK293T in 60-mm dishes transfected with indicated plasmids for 24 h, followed by MG132 (10 μM) treatment for 6 h. (G) The ubiquitination of endogenous IRF1 was analyzed by IP and immunoblot in the WT and *Xaf1*^−/−^ PMs in 10-cm dishes infected with PR8 (MOI 0.1) for 8 h, followed by MG132 (10 μM) treatment for 4 h. (H) Working model of this study. As an identified ISG, XAF1 is regulated by transcription factor IRF1, which in turn interacts with and stabilizes IRF1 by antagonizing CHIP-mediated degradation. Then, IRF1 triggers downstream IRF1-dependent IFN-I and antiviral ISGs production. This positive feedback regulatory loop of XAF1 and IRF1 promotes the host antiviral innate immunity. (A–G) Data are representative results of three independent experiments.

We next explored the mechanism by which XAF1 inhibits IRF1 degradation. It has been reported that XAF1 interacts with IRF1 and inhibits IRF1 degradation during tumor therapy (19). We confirmed the XAF1-IRF1 interaction by exogenous expression of XAF1 and IRF1 in HEK293T cells (Fig. 9A and B). The Mf2 and NLS region of IRF1 mediated the interaction between XAF1-IRF1 in the nucleus (Fig. 9C and D). E3 ligase CHIP mediates the ubiquitination and degradation of IRF1 (47). Our coimmunoprecipitation analysis for XAF1, IRF1, and CHIP expressed in HEK293T cells showed that the overexpression of XAF1 significantly inhibited the IRF1-CHIP interaction and restored exogenous IRF1 expression (Fig. 9E). Further ubiquitination analysis in the IRF1-overexpressed HEK293T cells showed that the overexpression of CHIP significantly promoted IRF1 K48 ubiquitination, while overexpression of XAF1 significantly inhibited CHIP-mediated K48 ubiquitination of IRF1 (Fig. 9F). Endogenous ubiquitination analysis in the PMs infected with PR8 revealed that knockout of XAF1 enhanced IRF1 ubiquitination and reduced the IRF1 protein level (Fig. 9G). These results indicated that XAF1 was essential for stabilizing IRF1 protein during viral infection. XAF1 inhibited the K48 ubiquitination and degradation of IRF1 by antagonizing CHIP-IRF1 interaction.

Our study has described a positive feedback loop between IRF1 and XAF1, which plays an important role during the host fighting against invaded RNA viruses independent of apoptosis (Fig. 9H). IRF1 induces XAF1 expression transcriptionally, and XAF1 stabilizes the IRF1 protein posttranslationally. IRF1 transcriptionally induced by the phosphorylated STAT1 and stabilized by XAF1 further facilitates the induction of the IRF1 target genes such as *DDX58*, *DDX60*, *MX1*, and *OAS2* during viral infection (Fig. 9H). These IRF1 target genes directly inhibit viral replication and positively regulate the RIG-I signaling pathway to induce more IFN-I in host antiviral innate immunity against RNA viruses.

## DISCUSSION

In the present study, we have provided evidence that XAF1 stabilizes IRF1 proteins and facilitates IRF1-mediated host defense against RNA viruses in an apoptosis-independent manner. XAF1 is considered a proapoptotic factor that suppresses tumorigenesis (17, 19), while we have shown that XAF1 plays a positive role in host antiviral innate immunity as many other antiviral ISGs do. XAF1 is a potent IRF1 stabilizer that promotes the induction of antiviral IRF1 target genes, suggesting that XAF1-IRF1 interaction is broadly beneficial for the host fighting against invading RNA viruses such as influenza, DENV, ZIKV, and SARS-CoV-2.

Mammalian IRF1 proteins are predominantly localized in the nucleus (26), allowing more rapid induction of antiviral genes and responses to viral infections. Basal expression of IRF1 drives intrinsic hepatocytes resistant to multiple RNA viruses, including HAV, HCV, DENV, and ZIKV (29). About 260 genes expressed constitutively in human respiratory epithelial cells are under the control of IRF1 (45). XAF1 was initially identified as a nuclear protein that binds and antagonizes the anticaspase activity of XIAP (48). Recently, studies further indicate that XAF1 colocalizes with the TFs, including p53 and IRF1 in the nucleus (17, 19). Therefore, it is possible that the interaction between XAF1 and IRF1 in the nucleus keeps low levels of expression of IRF1 target genes such as *MX1* and *OAS2* and maintains a basal antiviral state at the physiological condition. XAF1 and IRF1 form a positive feedback loop in the virus-infected cells to facilitate antiviral innate immunity under pathophysiological conditions.

We have shown that the expression of XAF1 is robustly and lastingly upregulated in the host cells infected by numerous RNA viruses *in vitro* and *in vivo*. Although the expression patterns of the XAF1 protein are different in the human cells such as PBMCs and A549 cells, XAF1A is the most dominant isoform. Viral infection activates NF-κB, MAPK, and JAK-STAT1/2 signaling, which may synergistically induce the IRF1 transcription (15, 39, 44). Our results suggest that IRF1 binds to the promoter region of *XAF1* and drives *XAF1* transcription dramatically. Given the rapid turnover of *IRF1* mRNA and IRF1 proteins, XAF1 has more potential than IRF1 to be a sensitive and reliable diagnostic marker of viral infectious diseases. Our data and other clinical data sets have suggested that elevated XAF1 expression is positively correlated with the SARS-CoV, MERS-CoV, and SARS-CoV-2 infection in the lungs (49, 50) as well as the symptoms of the COVID-19 and influenza patients (51).

The IRF-E (consensus sequence: AANNGAAA) and the ISRE (consensus sequence: GAAANNGAAA) are extremely similar (26). IRF-E could be regarded as a shortened version of ISRE. IRF1 binds to “GAAA” sequences within IRF-E and ISRE, while STAT1-STAT2-IRF9 (known as ISGF3) complex only recognizes the ISRE, which suggests that IRF1 can drive gene transcription independent of STAT1 and also regulate a larger range of ISGs than ISGF3. Our gene set enrichment analysis (GSEA) analysis results show that IRF1 target genes, but not STAT1 target genes, were significantly induced in the ZIKV-infected monkey PBMCs and VSV/SeV-infected mouse BMDMs. We selected and confirmed several IRF1 target genes (*DDX58*, *DDX60*, *MX1*, and *OAS2*), which were differentially expressed in the PR8-infected, 3p-hpRNA-transfected, or IFN-β-treated WT and *Xaf1^−/−^* macrophages or mice lungs. These IRF1 target genes indirectly or directly inhibit viral replication by targeting the viral life cycle or enhancing IFN-I-dependent antiviral immunity. IRF1 is more conserved than IFN-I and STAT1 (26), suggesting that IRF1-dependent antiviral immunity plays more important roles in the species without IFN-I signaling. Moreover, IRF1 target genes robustly and rapidly respond to viral infection, which is essential for host defense at the early stage. However, the IRF1 protein is extremely unstable and has a rapid turnover rate, which may limit its antiviral effect. Our study has provided a novel factor that stabilizes IRF1 protein and maintains IRF1-dependent antiviral immunity.

Transcriptional induction of *IRF1* mRNA is insufficient to accumulate the IRF1 proteins and maintain IRF1-dependent antiviral immunity. We have shown that the knockout of XAF1 significantly reduced the half-life of IRF1 proteins in the PR8-infected macrophages. IRF1 protein has a rapid turnover rate with a short half-life, and the K48-linked ubiquitination determines IRF1 stability (38, 39, 52–54). Several ubiquitin E3 ligases, including CHIP, have been shown to mediate the ubiquitin-proteasome system-dependent degradation of IRF1 via ubiquitination (40, 55). Our results indicate that knockout of XAF1 increased the interaction between the K48-ubiquitin chain and IRF1, and overexpression of XAF1 antagonizes the CHIP-IRF1 interaction. Therefore, in addition to inducing IRF1 transcription during viral infection, we have outlined an alternative pathway to enhance IRF1-dependent antiviral immunity.

In conclusion, we have demonstrated that XAF1 and IRF1 form a positive feedback loop to facilitate the host defense against numerous RNA viruses. Infection with RNA viruses upregulates XAF1 expression by inducing its master TF IRF1, and the elevated XAF1 stabilizes the IRF1 protein to induce more antiviral IRF1 target genes. Although we have described the protective role of the XAF1-IRF1 positive feedback loop during the host’s fight against several RNA viruses, further studies are required to elucidate the function of XAF1 when the host is challenged with other emerging RNA viruses, including SARS-CoV-2 and DNA viruses such as Herpes simplex virus and Adenovirus.

## MATERIALS AND METHODS

### Mice.

*Xaf1*^−/−^ mice (C57BL/6N background) were generated by Cyagen Biosciences (Suzhou, China). Briefly, a pair of single guide RNAs (sgRNAs) 5′-TACAAGTTAGCTAGGGCTGTT GG-3′ and 5′-TTCCGCTGTTCCAACGTGGTTGG-3′ were designed to generate a 4.4 kb chromosomal deletion on *Xaf1* (Gene ID: 327959). Mice genotyping primer sequences were 5′-GATGGAATGGGTTGGCAGCGTTC-3′ (F1), 5′-CTCCTTGCACACTCATGGGATTG-3′ (R1), and 5′-GTACCAGGGCAA CAGGCAACTTTC-3′ (R2). PCR product sizes were 635 bp (F1+R1, WT mice) and 910 bp (F1+R2, *Xaf1*^−/−^ mice). The WT C57BL/6N mice were ordered from Vital River Laboratory Animal Technology (Beijing, China). All the mice were maintained in the specific pathogen-free (SPF) environment at Suzhou Institute of Systems Medicine (ISM) under a controlled temperature (25°C) and a 12 h day-night cycle. All animal experiments were conducted according to the US National Institutes of Health Guide for the Care and Use of Laboratory Animals and approved by the Animal Service Center of ISM (ISM-IACUC-0011-R).

### Reagents.

Primary antibodies specific for IgG (No. 2729), GAPDH (No. 5174), XAF1 (No. 13805), IRF1 (No. 8478 and 79132), RIG-I (No. 3743), p-TBK1 (No. 5483), TBK1 (No. 3504), p-IRF3 (No. 29047), IRF3 (No. 4302), p-STAT1 (No. 9167), STAT1 (No. 14994), p-ERK1/2 (No. 9101), ERK1/2 (No. 4695), p-JNK (No. 4668), JNK (No. 9252S), p-p38 (No. 9215), p38 (No. 9212), p-p65 (No. 3033), p65 (No. 6956), HA-Tag (6E2)-HRP (No. 2999), Myc-Tag-HRP (No. 2040), and K48-Ub-HRP (No. 12805) were purchased from Cell Signaling Technology (Danvers, MA, USA). HRP-linked secondary antibodies specific for rabbit IgG and mouse IgG were also from Cell Signaling Technology. Primary antibodies against α-Tubulin (No. T5168), the anti-FLAG M2 Affinity Gel (No. A2220), and anti-HA-Agarose (No. A2095) were from Sigma (St.Louis, MO, USA). NF-κB inhibitor BAY11-7082, MAPK pathways inhibitors, including SB203580, PD98059 and SP600125, and JAK Inhibitor INCB018424 were purchased from InvivoGen (San Diego, CA).

### Cell culture.

Human embryonic kidney 293T (HEK293T) cells, human nonsmall-cell lung cancer A549, and Madin-Darby canine kidney (MDCK) cells were purchased from American Type Culture Collection (ATCC) and were cultured in Dulbecco's modified Eagle's medium (DMEM; GIBCO), supplemented with 10% heat-inactivated fetal bovine serum (FBS; GIBCO), 100 units/mL penicillin, and 100 μg/mL streptomycin (1% P/S; GIBCO), at 37°C and 10% CO_2_. For mouse primary peritoneal macrophage (PM) preparation, C57BL/6N mice (male, 6–8 weeks old) were intraperitoneally injected with a 3% fluid thioglycolate medium. Three days later, peritoneal exudate cells were collected and incubated. Then, 2 h later, the medium was replaced, and the adherent monolayer cells were PMs. PMs were cultured in RPMI 1640 medium (GIBCO) supplemented with 10% FBS and 1% P/S. To obtain human PBMCs, whole blood from separate healthy donors diluted with PBS (4 mL) was gently added above 3 mL of Ficoll-Paque media (GE Healthcare) in a Falcon tube and centrifuged with a density gradient. Four layers were formed, and PBMCs in the second layer were gently removed using a pipette and then added to PBS to wash off the remaining platelets. PBMCs were cultured in the RPMI 1640 medium supplemented with 10% FBS and macrophage colony-stimulating factor (M-CSF; R&D Systems) for 5 to 7 days at 37°C along with 10% CO_2_. The PBMCs used in the time-course experiments were from the same donor, and the PBMCs per replicate in each group were from separate donors. The use of PBMCs conforms to the institutional guidelines of Zhejiang University (IRB-2021-028).

### Virus infection, TCID_50_ assay, and PR8-luciferase assay.

Cells were infected with PR8, WSN, PR8-Luc, VSV, or VSV-GFP with the indicated multiplicity of infection (MOI) and time. The IAV viruses were diluted in RPMI 1640 or DMEM containing 0.5 μg/mL tosyl-l-phenylalanine chloromethyl ketone (TPCK)-trypsin (Sigma) and infected the cells for 1 h. These cells were washed with PBS and grown in the cell culture medium. IAV was propagated in the MDCK cells, and the viral titer was measured by 50% tissue culture infectious dose (TCID_50_) assay in the MDCK cells, as described previously (56). For *in vivo* PR8 infection, age- (8–10 weeks old) and sex-matched mice were anesthetized with 1% pentobarbital sodium, and the nasal cavity was dripped with 100 PFU of PR8. For the TCID_50_ assay, the IAV virus sample was diluted in virus growth medium (DMEM containing 1 μg/mL PCK-trypsin, 0.3% BSA, 1% P/S, and 25 mM HEPES buffer) across a 96-well tissue culture plate with MDCK cells. Then, 2 h later, the medium was replaced by the fresh medium, and the cells were incubated for up to 48 to 72 h. For the PR8-luciferase assay, cells were infected by the PR8-Luc virus at the indicated MOI and time points. The supernatants were collected, and the renilla luciferase activity was measured using the *Renilla* luciferase assay system (Promega) according to the manufacturer's instructions.

### ZIKV infection *in vitro* and *in vivo*.

ZIKV (GZ01/2016; GenBank accession no. KU820898) was propagated in the Aedes albopictus C6/36 cells and titrated by plaque assay on BHK-21 cells (57). A549 cells were infected with ZIKV at the indicated MOI for 24 h, total RNA was extracted from the ZIKV-infected cells for viral RNA measurement, and supernatant viruses were collected for plaque assay. For ZIKV infection in rhesus monkeys, the experiments were designed as described previously (58). Briefly, four healthy 5-year-old rhesus monkeys (5–6 kg) were screened by ELISA and showed IgG antibodies negative against ZIKV, DENV, JEV, and YFV. Rhesus monkeys were subcutaneously injected with 10^5^ PFU of ZIKV. Blood samples were collected 1 day before inoculation and 1, 8, and 14 days after inoculation. PBMCs were isolated from whole blood samples by gradient centrifugation using Ficoll-Plaque. Total RNA of PBMCs was extracted with a PureLink RNA minikit (Life Technologies) for transcriptome analysis.

### Plasmid construction.

Human *XAF*1 (Gene ID: 54739) and IRF1 (Gene ID: 3659) ORFs with indicated tags were PCR-amplified using HEK293T cDNA as the templates, followed by insertion into a FG-EH-DEST2-PGK-Puro-WPRE (FGEH) empty vector. IRF1 truncated mutants HA-ΔNLS and HA-ΔMf2 were cloned into the FGEH vector for interacting domain mapping. The GFP-tagged IRF1, IRF1-NLS, and IRF1-Mf2 were cloned into the FGEH vector for colocalization assay. Promoter reporter vectors containing the promoters of human *XAF1* (Gene ID: 54739), *DDX58* (Gene ID: 23586), *MX1* (Gene ID: 4599), *OAS2* (Gene ID: 4939), or *DDX60* (Gene ID: 55601) were generated by PCR amplification using the HEK293T genome as a template, followed by insertion into the pGL4.17 vector (Promega). Mutant promoter-reporter plasmids were constructed by the Muta-direct TM kit (Sbsgene). The IRF1 overexpression plasmid was kindly provided by Professor Genhong Cheng (University of California, Los Angeles, CA). HA-K48 Ub and CHIP plasmids were gifted by Dr. Jun Cui (Sun Yat-Sen University, China). All constructs were confirmed by Sanger DNA sequencing, and all the amplification primers were available upon request.

### Generation of XAF1-stably overexpressing, *IRF1^–/–^*, and *XAF1^–/–^* A549 cells.

Human Flag-XAF1-expressing plasmids with an FGEH backbone were transfected into the A549 cells, and these cells were selected by 2 μg/mL puromycin (InvivoGen) after 48 h transfection. After 2 weeks of selection, the population cells were cultured in the medium with 0.5 μg/mL puromycin, and the expression level of XAF1 was confirmed by immunoblot analysis. *IRF1^−/−^* A549 cells were generated using CRISPR/Cas9 technology. sgRNAs targeting *IRF1* (5′-CACCGTCTAGGCCGATACAAAGCAG-3′ and 5′-AAACCTGCTTTGTATCGGCCTAGAC-3′) and sgRNAs targeting *XAF1* (5′-CACCGAAGCTTGCAGTG CTCCTCCA-3′ and 5′-AAACTGGAGGAGCACTGCA AGCTTC-3′) were cloned into the lentiCRISPRv2 vectors (Addgene no. 52961). The HEK293T cells were transfected with *IRF1* or *XAF1* sgRNAs lentiCRISPRv2, pLS3, pLS4, and pLS5 plasmids to generate Lenti-CRISPR virus particles. Viral supernatants were harvested after 48 h transfection and used to infect WT A549 cells. Infected cells were selected with 2 μg/mL puromycin at 48 h postinfection. The selected cells were cultured in the medium with 0.5 μg/mL puromycin for 14 d, and the expression level of IRF1 or XAF1 was confirmed by immunoblot analysis.

### Cell transfection and stimulation.

The plasmids were transfected into HEK293T cells using Polyethylenimine (Polysciences) and A549 cells using Lipofectamine 2000 (Invitrogen). The siRNA control (5′-UUCUCCGAACGUGUCACGUTT-3′) and the siRNA (5′-GCUCCUGAAAGGGAAAUCUTT-3′) targeting *XAF1* were delivered into PBMCs using INTERFERin (Polyplus-transfection) according to the manufacturer’s instruction. Cells were treated with MG132 (10 mM; Sigma), CHX (100 mg/mL; Sigma), and recombinant IFN-α (500U/mL; PBL Assay Science) or IFN-β (100U/mL or 500U/mL; PBL Assay Science) at indicated concentration and time points.

### Immunoblot and immunoprecipitation (IP).

For immunoblot analysis, cells were lysed using cold radioimmunoprecipitation assay (RIPA) lysis buffer (Beyotime Biotechnology) supplemented with a complete protease inhibitor cocktail (Roche) and the phosphatase inhibitor PhosSTOP (Roche) according to the manufacturer's instructions. Protein concentrations of the extracts were measured using a bicinchoninic acid (BCA) assay (Beyotime Biotechnology) and equalized with lysis buffer. Equal amounts of the extracts were loaded and subjected to SDS-PAGE, transferred onto polyvinylidene difluoride (PVDF) membranes (Millipore), and blotted with the chemiluminescence horseradish peroxidase (HRP) substrate (Millipore). For IP, cells were lysed using lysis buffer (50 mM Tris-HCl, pH 7.5, 150 mM NaCl, 1 mM EDTA, and 1% Triton X-100) and a complete protease inhibitor cocktail. Extracts were incubated overnight with FLAG M2 Affinity Gel, anti-HA-Agarose, or appropriate antibodies plus protein A/G beads. Beads were washed, eluted, and then operated as immunoblot analysis.

### Immunofluorescence confocal microscopy.

HEK293T cells seeded on glass coverslips were transiently cotransfected with Flag-XAF1 and indicated GFP-tagged vectors for 24 h. Cells were washed briefly with PBS, fixed in 4% paraformaldehyde, permeabilized with 0.2% Triton X-100 in PBS, and blocked with 5% goat serum. For colocalization assays of XAF1 and IRF1, cells were incubated with anti-FLAG primary antibody and stained with the fluorescent secondary antibody. After 4′,6-diamidino-2-phenylindole (DAPI) incubation, fluorescent imaging was obtained with a LEICA TCA SP8 confocal microscope.

### RNA-sequencing (RNA-seq) and transcriptomes analysis.

For ZIKV-infected monkey PBMCs, the quality of the extracted RNA was evaluated by the Agilent Technologies 2100 Bioanalyzer, and RNA-seq libraries were constructed using an Illumina TruSeq stranded mRNA sample preparation kit according to the manufacturer's guidelines. High-throughput sequencing was performed using a Hiseq 10X platform with the paired-end 2 × 150-bp, dual-index format. For RNA-seq data analysis, first, trimmomatic was used to remove Illumina sequencing adapters within the raw reads of every sample and trim the low-quality bases of both reads ends. Second, the clean reads were aligned to the rhesus monkey genome (rheMac10) with STAR, and the alignment bam files were used as an HTSeq-count (command of the python package HTSeq) input to obtain the gene read counts. The Benjamini Hochberg method was used to adjust the generated *P*-value to control the false discovery rate (FDR). Genes with a FDR < 0.05 and fold change >2 were identified as significantly differentially expressed genes (DEGs) under the conditions of biological replication of two RNA sequences. For microarray analysis of IAV patients, the microarray raw data (accession no. GSE101702) were downloaded from GEO, and the XAF1 expression value was obtained using GEO2R. For RNA-seq analysis of the ZIKV patient and the SARS-COV-2 patients, the RNA-seq raw data were from GEO (accession no. GSE123541 and GSE167000, respectively). For VSV- and SeV-infected BMDMs, reads were mapped to the Ensembl GRCm38.p6 reference genome and normalized by the RNA-seq analysis program of CLC Genomics Workbench 12.0 (Qiagen). Fold changes and *P*-values of genes between groups with different treatments (triplication for each treatment) were calculated by the differential expression program in CLC genomic workbench 12.0 with a trimmed mean M-values (TMM) normalization.

### RNA isolation and real-time qRT-PCR.

Total RNA was isolated from cells or lung tissues using TRIzol reagent (ThermoFisher Scientific) according to the manufacturer’s directions. cDNA was synthesized using the PrimeScript RT Master Mix (TaKaRa). TB Green Premix *Ex Taq* (Tli RNaseH Plus) was used for qRT-PCR amplification on a Roche LightCycler 480 II system. qRT-PCR primer sequences for target genes were listed in Table S1.

### DNA pulldown.

Cells were lysed using lysis buffer (50 mM Tris-HCl, pH 7.5, 150 mM NaCl, 1 mM EDTA, and 1% Triton X-100) supplemented with the complete protease inhibitor cocktail. Extracts were incubated overnight with biotinylated DNA probes and then with Pierce streptavidin agarose for 2 h (Production no. 20349, ThermoFisher). Beads were washed, eluted, and then operated according to immunoblot analysis. Biotinylated DNA probe sequences are listed in Table S2.

### ELISA and dual-luciferase reporter assay.

The IFN-α and IFN-β in cell supernatant or lung homogenate were measured by mouse IFN alpha ELISA kits and mouse IFN beta ELISA kits (PBL Assay Science) according to manufacturer's instructions, respectively. The promoter-reporter plasmid, renilla luciferase reporter (pRL-TK-Luc) plasmid, and other indicated plasmids were cotransfected into HEK293T cells. At 24 h posttransfection, the cells were lysed by passive lysis buffer, and the luciferase activity of promoter reporters was measured by the dual-luciferase reporter assay system (Promega) according to the manufacturer's instructions.

### Pathological examination and immunohistochemistry.

Lung tissues from control or virus-infected mice were collected, fixed in 4% paraformaldehyde in PBS, embedded into paraffin, sectioned at 4 μm, stained by H&E, and evaluated by light microscopy for pathological injury. The degree of lung injury was assessed by a pulmonary pathologist using optical microscopy at ×200 magnification on five random fields. The histological score of lung injury was assessed as described in previous study (59). Briefly, the pulmonary pathologist evaluated lung sections in a randomized and blinded manner to assess the histopathological grade of acute lung injury using the following parameters: interstitial edema, alveolar edema, hemorrhage, cellular infiltration, and hyaline membrane formation. The severity of the slides was graded on a four-point scale, as follows: 0, absent; 1, gentle; 2, moderate; and 3, severe. Neutrophil marker myeloperoxidase (MPO) was detected by immunohistochemistry in mouse lung tissues. Briefly, 4 μm paraffin sections of lung tissues were fixed with xylene, quenched with 3% H_2_O_2,_ and blocked with goat serum. The sections were treated with anti-MPO primary antibody (ab9535; Abcam) overnight. Then, the slices were incubated with the HRP-conjugated secondary antibody. One hour later, 3,3' diaminobenzidine (DAB) was used as the substrate of peroxidase. The slides were stained with hematoxylin and observed by light microscopy.

### Detection of GFP by flow cytometry.

The cells were infected with VSV-GFP or SeV-GFP at the indicated MOI and time points. The infected cells were detached with trypsin (Gibco) at 37°C for 5 min, incubated with DMEM containing 10% FBS, and washed twice in PBS. Then the cells were transferred to PBS containing 8% formaldehyde. After incubating for 20 min at room temperature, the fixed cells were centrifuged at 1,500 g for 5 min, washed once in PBS, and resuspended in 500 μL of PBS. GFP fluorescence was detected by flow cytometry (Life Attune NxT; Thermo Fisher Scientific).

### Quantification and statistical analysis.

The number of experiment repetitions is shown in the figure legends. Statistical significance was performed by unpaired two-tailed Student's *t* test in GraphPad Prism 8 software. The statistical significance of *in vivo* survival data were plotted as a Kaplan-Meier survival curve and determined by the log-rank (Mantel-Cox) test. Differences were considered statistically significant when *P* < 0.05.

### Data availability.

The raw sequence data generated in this paper have been deposited in the Genome Sequence Archive in National Genomics Data Center, China National Center for Bioinformation/Beijing Institute of Genomics, Chinese Academy of Sciences, under accession number CRA004891 (Rhesus monkey PBMC) and CRA004996 (BMDMs) and are publicly accessible at https://ngdc.cncb.ac.cn/gsa.

## References

[1] Oboho IK, Tomczyk SM, Al-Asmari AM, Banjar AA, Al-Mugti H, Aloraini MS, Alkhaldi KZ, Almohammadi EL, Alraddadi BM, Gerber SI, Swerdlow DL, Watson JT, Madani TA. 2015. 2014 MERS-CoV outbreak in Jeddah–a link to health care facilities. N Engl J Med 372:846–854. 10.1056/NEJMoa1408636. 25714162 PMC5710730

[2] Gao GF. 2018. From “A”IV to “Z”IKV: attacks from Emerging and Re-emerging Pathogens. Cell 172:1157–1159. 10.1016/j.cell.2018.02.025. 29522735 PMC7126677

[3] Woolf SH, Chapman DA, Lee JH. 2021. COVID-19 as the leading cause of death in the United States. JAMA 325:123–124. 10.1001/jama.2020.24865. 33331845 PMC8553021

[4] Kawai T, Akira S. 2010. The role of pattern-recognition receptors in innate immunity: update on Toll-like receptors. Nat Immunol 11:373–384. 10.1038/ni.1863. 20404851

[5] Barbalat R, Ewald SE, Mouchess ML, Barton GM. 2011. Nucleic acid recognition by the innate immune system. Annu Rev Immunol 29:185–214. 10.1146/annurev-immunol-031210-101340. 21219183

[6] Goubau D, Deddouche S, Reis e Sousa C. 2013. Cytosolic sensing of viruses. Immunity 38:855–869. 10.1016/j.immuni.2013.05.007. 23706667 PMC7111113

[7] Wu J, Chen ZJ. 2014. Innate immune sensing and signaling of cytosolic nucleic acids. Annu Rev Immunol 32:461–488. 10.1146/annurev-immunol-032713-120156. 24655297

[8] Honda K, Takaoka A, Taniguchi T. 2006. Type I interferon [corrected] gene induction by the interferon regulatory factor family of transcription factors. Immunity 25:349–360. 10.1016/j.immuni.2006.08.009. 16979567

[9] Sadler AJ, Williams BR. 2008. Interferon-inducible antiviral effectors. Nat Rev Immunol 8:559–568. 10.1038/nri2314. 18575461 PMC2522268

[10] Schneider WM, Chevillotte MD, Rice CM. 2014. Interferon-stimulated genes: a complex web of host defenses. Annu Rev Immunol 32:513–545. 10.1146/annurev-immunol-032713-120231. 24555472 PMC4313732

[11] Verhelst J, Parthoens E, Schepens B, Fiers W, Saelens X. 2012. Interferon-inducible protein Mx1 inhibits influenza virus by interfering with functional viral ribonucleoprotein complex assembly. J Virol 86:13445–13455. 10.1128/JVI.01682-12. 23015724 PMC3503048

[12] Miyashita M, Oshiumi H, Matsumoto M, Seya T. 2011. DDX60, a DEXD/H box helicase, is a novel antiviral factor promoting RIG-I-like receptor-mediated signaling. Mol Cell Biol 31:3802–3819. 10.1128/MCB.01368-10. 21791617 PMC3165724

[13] Oshiumi H, Miyashita M, Okamoto M, Morioka Y, Okabe M, Matsumoto M, Seya T. 2015. DDX60 Is Involved in RIG-I-Dependent and Independent Antiviral Responses, and Its Function Is Attenuated by Virus-Induced EGFR Activation. Cell Rep 11:1193–1207. 10.1016/j.celrep.2015.04.047. 25981042

[14] Leaman DW, Chawla-Sarkar M, Vyas K, Reheman M, Tamai K, Toji S, Borden EC. 2002. Identification of X-linked inhibitor of apoptosis-associated factor-1 as an interferon-stimulated gene that augments TRAIL Apo2L-induced apoptosis. J Biol Chem 277:28504–28511. 10.1074/jbc.M204851200. 12029096

[15] Sun Y, Qiao L, Xia HH, Lin MC, Zou B, Yuan Y, Zhu S, Gu Q, Cheung TK, Kung HF, Yuen MF, Chan AO, Wong BC. 2008. Regulation of XAF1 expression in human colon cancer cell by interferon beta: activation by the transcription regulator STAT1. Cancer Lett 260:62–71. 10.1016/j.canlet.2007.10.014. 18035482

[16] Zhang X, Yang W, Wang X, Zhang X, Tian H, Deng H, Zhang L, Gao G. 2018. Identification of new type I interferon-stimulated genes and investigation of their involvement in IFN-beta activation. Protein Cell 9:799–807. 10.1007/s13238-018-0511-1. 29427062 PMC6107486

[17] Lee MG, Han J, Jeong SI, Her NG, Lee JH, Ha TK, Kang MJ, Ryu BK, Chi SG. 2014. XAF1 directs apoptotic switch of p53 signaling through activation of HIPK2 and ZNF313. Proc Natl Acad Sci USA 111:15532–15537. 10.1073/pnas.1411746111. 25313037 PMC4217407

[18] Micali OC, Cheung HH, Plenchette S, Hurley SL, Liston P, LaCasse EC, Korneluk RG. 2007. Silencing of the XAF1 gene by promoter hypermethylation in cancer cells and reactivation to TRAIL-sensitization by IFN-beta. BMC Cancer 7:52. 10.1186/1471-2407-7-52. 17376236 PMC1845166

[19] Jeong SI, Kim JW, Ko KP, Ryu BK, Lee MG, Kim HJ, Chi SG. 2018. XAF1 forms a positive feedback loop with IRF-1 to drive apoptotic stress response and suppress tumorigenesis. Cell Death Dis 9:806. 10.1038/s41419-018-0867-4. 30042418 PMC6057933

[20] Pinto EM, Figueiredo BC, Chen W, Galvao HCR, Formiga MN, Fragoso M, Ashton-Prolla P, Ribeiro E, Felix G, Costa TEB, Savage SA, Yeager M, Palmero EI, Volc S, Salvador H, Fuster-Soler JL, Lavarino C, Chantada G, Vaur D, Odone-Filho V, Brugieres L, Else T, Stoffel EM, Maxwell KN, Achatz MI, Kowalski L, de Andrade KC, Pappo A, Letouze E, Latronico AC, Mendonca BB, Almeida MQ, Brondani VB, Bittar CM, Soares EWS, Mathias C, Ramos CRN, Machado M, Zhou W, Jones K, Vogt A, Klincha PP, Santiago KM, Komechen H, Paraizo MM, Parise IZS, Hamilton KV, Wang J, Rampersaud E, Clay MR, et al. 2020. XAF1 as a modifier of p53 function and cancer susceptibility. Sci Adv 6:eaba3231. 10.1126/sciadv.aba3231. 32637605 PMC7314530

[21] Long X, Li Y, Qi Y, Xu J, Wang Z, Zhang X, Zhang D, Zhang L, Huang J. 2013. XAF1 contributes to dengue virus-induced apoptosis in vascular endothelial cells. FASEB J 27:1062–1073. 10.1096/fj.12-213967. 23207547

[22] Fujita T, Sakakibara J, Sudo Y, Miyamoto M, Kimura Y, Taniguchi T. 1988. Evidence for a nuclear factor(s), IRF-1, mediating induction and silencing properties to human IFN-beta gene regulatory elements. EMBO J 7:3397–3405. 10.1002/j.1460-2075.1988.tb03213.x. 2850164 PMC454838

[23] Pine R, Decker T, Kessler DS, Levy DE, Darnell JE, Jr. 1990. Purification and cloning of interferon-stimulated gene factor 2 (ISGF2): ISGF2 (IRF-1) can bind to the promoters of both beta interferon- and interferon-stimulated genes but is not a primary transcriptional activator of either. Mol Cell Biol 10:2448–2457. 10.1128/mcb.10.6.2448-2457.1990. 2342456 PMC360601

[24] Sauerhering L, Kupke A, Meier L, Dietzel E, Hoppe J, Gruber AD, Gattenloehner S, Witte B, Fink L, Hofmann N, Zimmermann T, Goesmann A, Nist A, Stiewe T, Becker S, Herold S, Peteranderl C. 2020. Cyclophilin inhibitors restrict Middle East respiratory syndrome coronavirus via interferon-lambda in vitro and in mice. Eur Respir J 56:1901826. 10.1183/13993003.01826-2019. 32616594 PMC7331652

[25] Yoo JS, Sasaki M, Cho SX, Kasuga Y, Zhu B, Ouda R, Orba Y, de Figueiredo P, Sawa H, Kobayashi KS. 2021. SARS-CoV-2 inhibits induction of the MHC class I pathway by targeting the STAT1-IRF1-NLRC5 axis. Nat Commun 12:6602. 10.1038/s41467-021-26910-8. 34782627 PMC8594428

[26] Feng H, Zhang YB, Gui JF, Lemon SM, Yamane D. 2021. Interferon regulatory factor 1 (IRF1) and anti-pathogen innate immune responses. PLoS Pathog 17:e1009220. 10.1371/journal.ppat.1009220. 33476326 PMC7819612

[27] Shaw AE, Hughes J, Gu Q, Behdenna A, Singer JB, Dennis T, Orton RJ, Varela M, Gifford RJ, Wilson SJ, Palmarini M. 2017. Fundamental properties of the mammalian innate immune system revealed by multispecies comparison of type I interferon responses. PLoS Biol 15:e2004086. 10.1371/journal.pbio.2004086. 29253856 PMC5747502

[28] Schaper F, Kirchhoff S, Posern G, Koster M, Oumard A, Sharf R, Levi BZ, Hauser H. 1998. Functional domains of interferon regulatory factor I (IRF-1). Biochem J 335:147–157. 10.1042/bj3350147. 9742224 PMC1219763

[29] Yamane D, Feng H, Rivera-Serrano EE, Selitsky SR, Hirai-Yuki A, Das A, McKnight KL, Misumi I, Hensley L, Lovell W, Gonzalez-Lopez O, Suzuki R, Matsuda M, Nakanishi H, Ohto-Nakanishi T, Hishiki T, Wauthier E, Oikawa T, Morita K, Reid LM, Sethupathy P, Kohara M, Whitmire JK, Lemon SM. 2019. Basal expression of interferon regulatory factor 1 drives intrinsic hepatocyte resistance to multiple RNA viruses. Nat Microbiol 4:1096–1104. 10.1038/s41564-019-0425-6. 30988429 PMC6588457

[30] Reis LF, Ruffner H, Stark G, Aguet M, Weissmann C. 1994. Mice devoid of interferon regulatory factor 1 (IRF-1) show normal expression of type I interferon genes. EMBO J 13:4798–4806. 10.1002/j.1460-2075.1994.tb06805.x. 7957048 PMC395418

[31] Ruffner H, Reis LF, Naf D, Weissmann C. 1993. Induction of type I interferon genes and interferon-inducible genes in embryonal stem cells devoid of interferon regulatory factor 1. Proc Natl Acad Sci USA 90:11503–11507. 10.1073/pnas.90.24.11503. 8265581 PMC48012

[32] Tamura T, Yanai H, Savitsky D, Taniguchi T. 2008. The IRF family transcription factors in immunity and oncogenesis. Annu Rev Immunol 26:535–584. 10.1146/annurev.immunol.26.021607.090400. 18303999

[33] Carlin AF, Plummer EM, Vizcarra EA, Sheets N, Joo Y, Tang W, Day J, Greenbaum J, Glass CK, Diamond MS, Shresta S. 2017. An IRF-3-, IRF-5-, and IRF-7-independent pathway of Dengue viral resistance utilizes IRF-1 to stimulate type I and II interferon responses. Cell Rep 21:1600–1612. 10.1016/j.celrep.2017.10.054. 29117564 PMC5696617

[34] Zhou H, Tang YD, Zheng C. 2022. Revisiting IRF1-mediated antiviral innate immunity. Cytokine Growth Factor Rev 64:1–6. 10.1016/j.cytogfr.2022.01.004. 35090813

[35] Odendall C, Dixit E, Stavru F, Bierne H, Franz KM, Durbin AF, Boulant S, Gehrke L, Cossart P, Kagan JC. 2014. Diverse intracellular pathogens activate type III interferon expression from peroxisomes. Nat Immunol 15:717–726. 10.1038/ni.2915. 24952503 PMC4106986

[36] Motz C, Schuhmann KM, Kirchhofer A, Moldt M, Witte G, Conzelmann KK, Hopfner KP. 2013. Paramyxovirus V proteins disrupt the fold of the RNA sensor MDA5 to inhibit antiviral signaling. Science 339:690–693. 10.1126/science.1230949. 23328395

[37] Schmitz F, Heit A, Guggemoos S, Krug A, Mages J, Schiemann M, Adler H, Drexler I, Haas T, Lang R, Wagner H. 2007. Interferon-regulatory-factor 1 controls Toll-like receptor 9-mediated IFN-beta production in myeloid dendritic cells. Eur J Immunol 37:315–327. 10.1002/eji.200636767. 17273999

[38] Watanabe N, Sakakibara J, Hovanessian AG, Taniguchi T, Fujita T. 1991. Activation of IFN-beta element by IRF-1 requires a posttranslational event in addition to IRF-1 synthesis. Nucleic Acids Res 19:4421–4428. 10.1093/nar/19.16.4421. 1886766 PMC328629

[39] Feng H, Lenarcic EM, Yamane D, Wauthier E, Mo J, Guo H, McGivern DR, Gonzalez-Lopez O, Misumi I, Reid LM, Whitmire JK, Ting JP, Duncan JA, Moorman NJ, Lemon SM. 2017. NLRX1 promotes immediate IRF1-directed antiviral responses by limiting dsRNA-activated translational inhibition mediated by PKR. Nat Immunol 18:1299–1309. 10.1038/ni.3853. 28967880 PMC5690873

[40] Narayan V, Pion E, Landre V, Muller P, Ball KL. 2011. Docking-dependent ubiquitination of the interferon regulatory factor-1 tumor suppressor protein by the ubiquitin ligase CHIP. J Biol Chem 286:607–619. 10.1074/jbc.M110.153122. 20947504 PMC3013021

[41] Yoneyama M, Kikuchi M, Natsukawa T, Shinobu N, Imaizumi T, Miyagishi M, Taira K, Akira S, Fujita T. 2004. The RNA helicase RIG-I has an essential function in double-stranded RNA-induced innate antiviral responses. Nat Immunol 5:730–737. 10.1038/ni1087. 15208624

[42] Hornung V, Ellegast J, Kim S, Brzozka K, Jung A, Kato H, Poeck H, Akira S, Conzelmann KK, Schlee M, Endres S, Hartmann G. 2006. 5'-Triphosphate RNA is the ligand for RIG-I. Science 314:994–997. 10.1126/science.1132505. 17038590

[43] Takaoka A, Yanai H. 2006. Interferon signalling network in innate defence. Cell Microbiol 8:907–922. 10.1111/j.1462-5822.2006.00716.x. 16681834

[44] Wang J, Zhang W, Zhang Y, Chen Y, Zou B, Jiang B, Pang R, Gu Q, Qiao L, Lan H, Kung HF, Wong BC. 2009. c-Jun N-terminal kinase (JNK1) upregulates XIAP-associated factor 1 (XAF1) through interferon regulatory factor 1 (IRF-1) in gastrointestinal cancer. Carcinogenesis 30:222–229. 10.1093/carcin/bgn271. 19056926

[45] Panda D, Gjinaj E, Bachu M, Squire E, Novatt H, Ozato K, Rabin RL. 2019. IRF1 maintains optimal constitutive expression of antiviral genes and regulates the early antiviral response. Front Immunol 10:1019. 10.3389/fimmu.2019.01019. 31156620 PMC6529937

[46] Taniguchi T, Ogasawara K, Takaoka A, Tanaka N. 2001. IRF family of transcription factors as regulators of host defense. Annu Rev Immunol 19:623–655. 10.1146/annurev.immunol.19.1.623. 11244049

[47] Landré V, Pion E, Narayan V, Xirodimas DP, Ball KL. 2013. DNA-binding regulates site-specific ubiquitination of IRF-1. Biochem J 449:707–717. 10.1042/BJ20121076. 23134341

[48] Liston P, Fong WG, Kelly NL, Toji S, Miyazaki T, Conte D, Tamai K, Craig CG, McBurney MW, Korneluk RG. 2001. Identification of XAF1 as an antagonist of XIAP anti-Caspase activity. Nat Cell Biol 3:128–133. 10.1038/35055027. 11175744

[49] Gao X, Liu Y, Zou S, Liu P, Zhao J, Yang C, Liang M, Yang J. 2021. Genome-wide screening of SARS-CoV-2 infection-related genes based on the blood leukocytes sequencing data set of patients with COVID-19. J Med Virol 93:5544–5554. 10.1002/jmv.27093. 34009691 PMC8242610

[50] Park A, Harris LK. 2021. Gene expression meta-analysis reveals interferon-induced genes associated with SARS infection in lungs. Front Immunol 12:694355. 10.3389/fimmu.2021.694355. 34367154 PMC8342995

[51] Zhu L, Yang P, Zhao Y, Zhuang Z, Wang Z, Song R, Zhang J, Liu C, Gao Q, Xu Q, Wei X, Sun HX, Ye B, Wu Y, Zhang N, Lei G, Yu L, Yan J, Diao G, Meng F, Bai C, Mao P, Yu Y, Wang M, Yuan Y, Deng Q, Li Z, Huang Y, Hu G, Liu Y, Wang X, Xu Z, Liu P, Bi Y, Shi Y, Zhang S, Chen Z, Wang J, Xu X, Wu G, Wang FS, Gao GF, Liu L, Liu WJ. 2020. Single-cell sequencing of peripheral mononuclear cells reveals distinct immune response landscapes of COVID-19 and influenza patients. Immunity 53:685–696. 10.1016/j.immuni.2020.07.009. 32783921 PMC7368915

[52] Nakagawa K, Yokosawa H. 2000. Degradation of transcription factor IRF-1 by the ubiquitin-proteasome pathway. The C-terminal region governs the protein stability. Eur J Biochem 267:1680–1686. 10.1046/j.1432-1327.2000.01163.x. 10712599

[53] Gao Z, Li Y, Wang F, Huang T, Fan K, Zhang Y, Zhong J, Cao Q, Chao T, Jia J, Yang S, Zhang L, Xiao Y, Zhou JY, Feng XH, Jin J. 2017. Mitochondrial dynamics controls anti-tumour innate immunity by regulating CHIP-IRF1 axis stability. Nat Commun 8:1805. 10.1038/s41467-017-01919-0. 29180626 PMC5703766

[54] Remoli AL, Sgarbanti M, Perrotti E, Acchioni M, Orsatti R, Acchioni C, Battistini A, Clarke R, Marsili G. 2020. IkappaB kinase-epsilon-mediated phosphorylation triggers IRF-1 degradation in breast cancer cells. Neoplasia 22:459–469. 10.1016/j.neo.2020.07.004. 32784074 PMC7419274

[55] Gao B, Wang Y, Xu W, Li S, Li Q, Xiong S. 2013. Inhibition of histone deacetylase activity suppresses IFN-gamma induction of tripartite motif 22 via CHIP-mediated proteasomal degradation of IRF-1. J Immunol 191:464–471. 10.4049/jimmunol.1203533. 23729439

[56] Karakus U, Crameri M, Lanz C, Yanguez E. 2018. Propagation and Titration of Influenza Viruses. Methods Mol Biol 1836:59–88. 10.1007/978-1-4939-8678-1_4. 30151569

[57] Li C, Deng YQ, Wang S, Ma F, Aliyari R, Huang XY, Zhang NN, Watanabe M, Dong HL, Liu P, Li XF, Ye Q, Tian M, Hong S, Fan J, Zhao H, Li L, Vishlaghi N, Buth JE, Au C, Liu Y, Lu N, Du P, Qin FX, Zhang B, Gong D, Dai X, Sun R, Novitch BG, Xu Z, Qin CF, Cheng G. 2017. 25-Hydroxycholesterol protects host against Zika virus infection and its associated microcephaly in a mouse model. Immunity 46:446–456. 10.1016/j.immuni.2017.02.012. 28314593 PMC5957489

[58] Li XF, Dong HL, Huang XY, Qiu YF, Wang HJ, Deng YQ, Zhang NN, Ye Q, Zhao H, Liu ZY, Fan H, An XP, Sun SH, Gao B, Fa YZ, Tong YG, Zhang FC, Gao GF, Cao WC, Shi PY, Qin CF. 2016. Characterization of a 2016 clinical isolate of Zika virus in non-human primates. EBioMedicine 12:170–177. 10.1016/j.ebiom.2016.09.022. 27693104 PMC5078627

[59] Cypel M, Rubacha M, Yeung J, Hirayama S, Torbicki K, Madonik M, Fischer S, Hwang D, Pierre A, Waddell TK, de Perrot M, Liu M, Keshavjee S. 2009. Normothermic ex vivo perfusion prevents lung injury compared to extended cold preservation for transplantation. Am J Transplant 9:2262–2269. 10.1111/j.1600-6143.2009.02775.x. 19663886

