# Supplementary material for: XAF1 Protects Host against Emerging RNA Viruses by Stabilizing IRF1-Dependent Antiviral Immunity
Source: J Virol. 2022 Aug 16;96(17):e00774-22. doi: 10.1128/jvi.00774-22 (PMC9472610; doi:10.1128/jvi.00774-22)
Supplement: Supplemental file 1 — Fig. S1 to Fig. S7. Download jvi.00774-22-s0001.pdf, PDF file, 12.4 MB [file jvi.00774-22-s0001.pdf]

Supplementary Figures for

XAF1 protects host against emerging RNA viruses by stabilizing IRF1-dependent antiviral immunity

Han Y. et al, Fig. S1

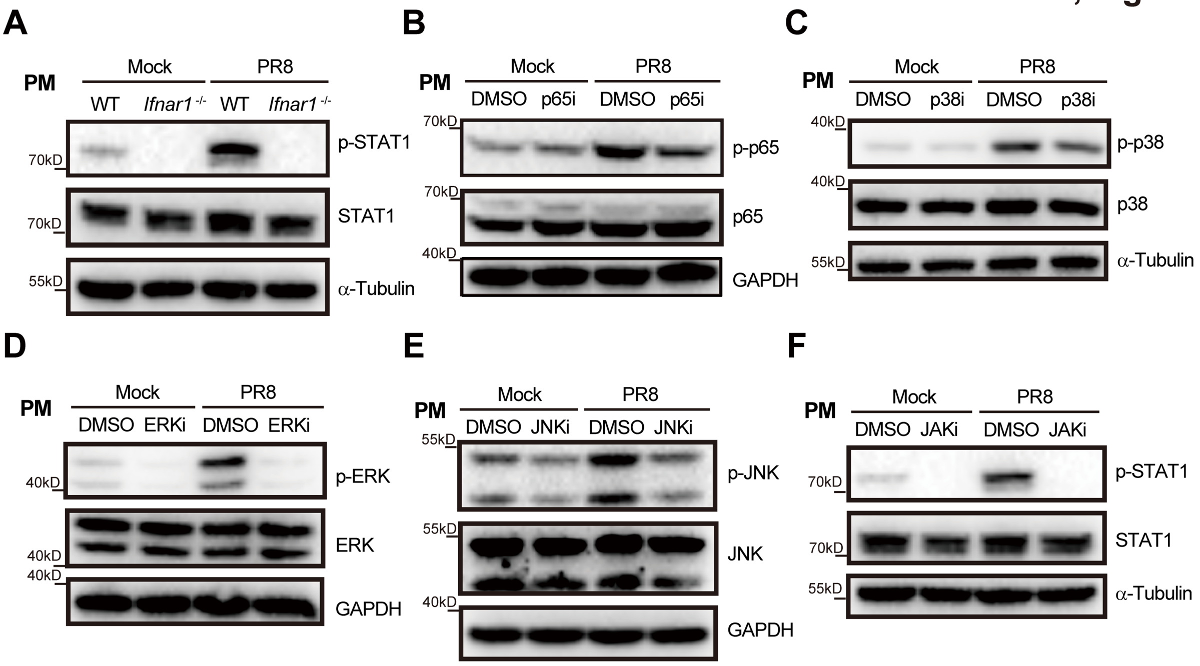

Fig. S1. Verification of the *Ifnar1*$^{-/-}$ PMs and kinase inhibitors. (A) Immunoblot analysis of indicated proteins in PMs from the WT and *Ifnar1*$^{-/-}$ mice infected with PR8 (MOI 0.1) for 4 h. (B-F) Immunoblot analysis of indicated proteins in the PMs pretreated for 1 h with the indicated inhibitors, followed by PR8 infection (MOI 0.1) for 4 h. All data are representative results of three independent experiments.

Han Y. et al, Fig. S2

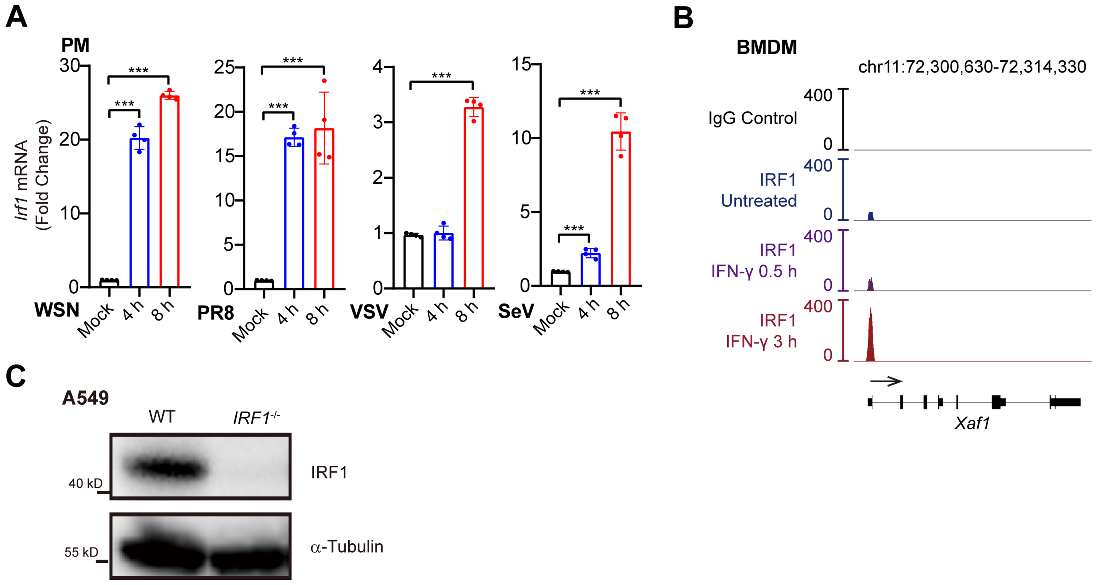

Fig. S2. Induction of IRF1 binding to the promoter of *XAF1* and verification of the *IRF1*$^{-/-}$ A549 cells. (A) qRT-PCR analysis of *Irf1* mRNA expression in the PMs infected with WSN (MOI 0.1), PR8 (MOI 0.1), VSV (MOI 1), and SeV(MOI 0.1) for the indicated time points. (B) BMDMs were treated with 400 U/ml IFN-γ for the indicated time points, IRF1 ChIP-Seq data were analyzed, and the IRF1 binding regions in the *Xaf1* promoter are shown. The IRF1 ChIP-Seq raw data were downloaded from GEO (accession no. GSE77886). (C) Immunoblot analysis of IRF1 protein in the WT and *IRF1*$^{-/-}$ A549 cells. Data of (A) from three independent experiments are presented as mean ± SD; ***P < 0.001 indicates significant difference by unpaired Student's *t*-test; Data of (C) are representative results of three independent experiments.

Han Y. et al, Fig. S3

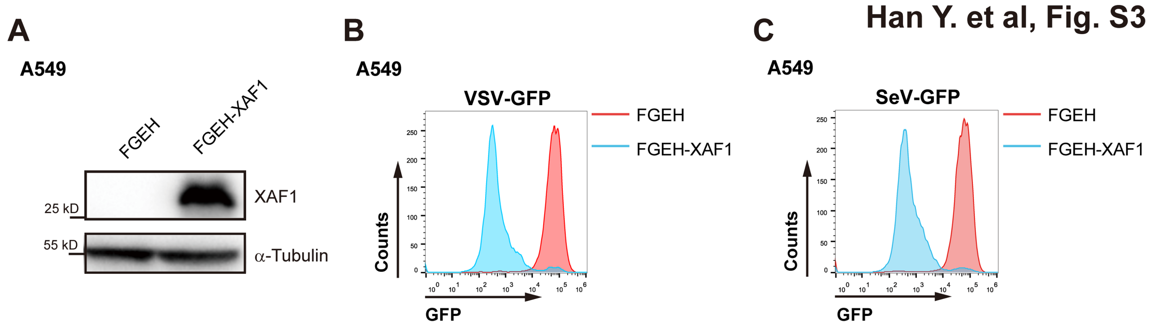

Fig. S3. XAF1 protects host against VSV and SeV. (A) Immunoblot analysis of XAF1 expression in the A549 cells stably overexpressing XAF1. (B and C) Flow cytometry analysis of GFP fluorescence intensity in the FGEH and FGEH-XAF1-expressing A549 cells infected with VSV-GFP (MOI 1) for 8 h (B) or SeV-GFP (MOI, 0.1) for 8 h (C). All data are representative results of three independent experiments.

Han Y. et al, Fig. S4

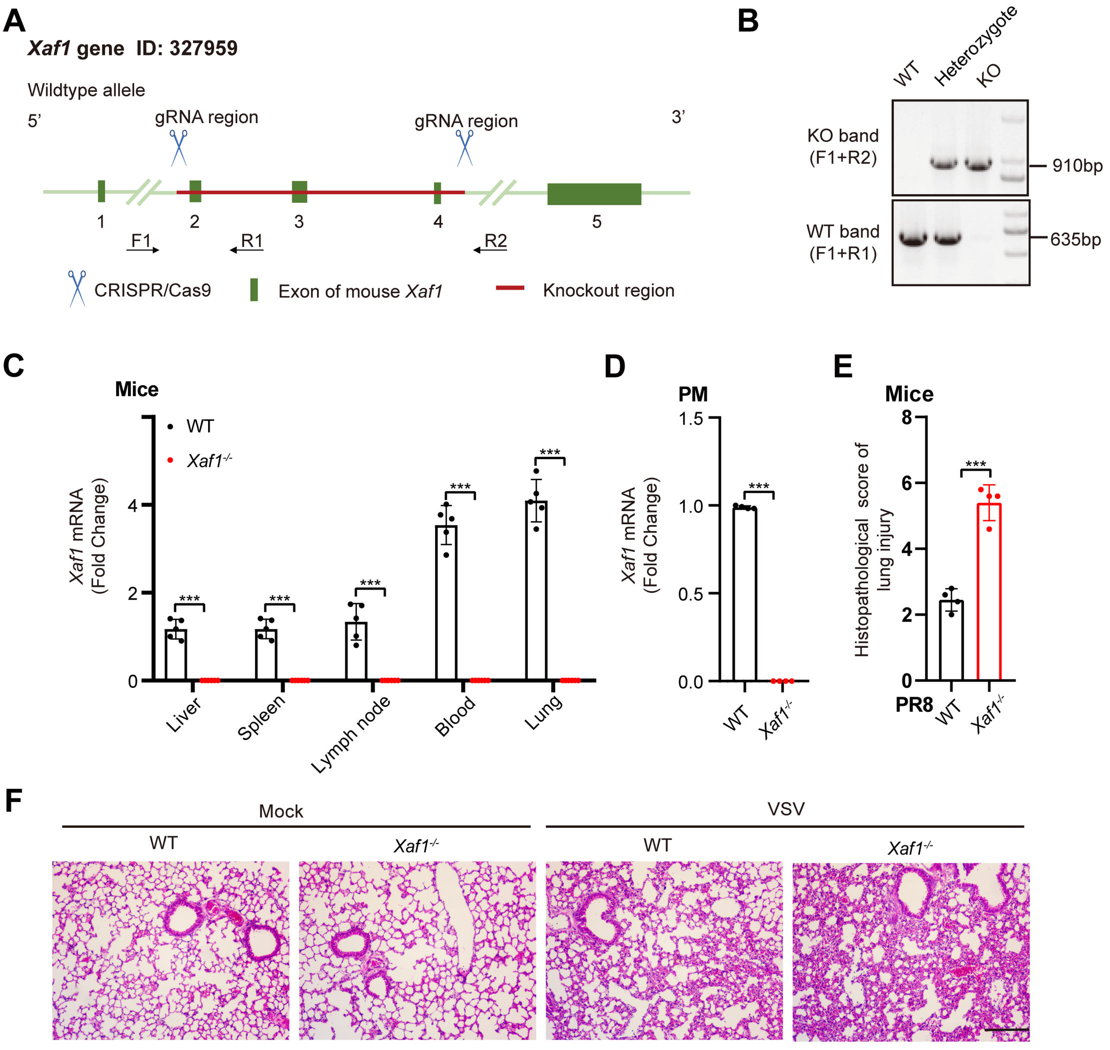

Fig. S4. Knockout of XAF1 facilitates RNA virus infection *in vivo*. (A) The *Xaf1* was deleted in the C57BL/6N background mouse chromosome by CRISPR/Cas9 targeting strategy. (B) Genotyping examinations of the WT, *Xaf1*$^{+/-}$, and *Xaf1*$^{-/-}$ mice by PCR. (C) qRT-PCR analysis of *Xaf1* mRNA level in the livers, spleens, lymph nodes, peripheral blood, and lungs from the WT (n=5) and *Xaf1*$^{-/-}$ (n=6) mice. (D) qRT-PCR analysis of *Xaf1* mRNA level in the WT and *Xaf1*$^{-/-}$ PMs. (E) Histopathological evaluation of the lung injury in the lung

sections from the WT and *Xaf1*$^{-/-}$ mice infected with PR8 (100 pfu) intranasally for 5 d. (F) Histopathological examination of the lung sections from the WT and *Xaf1*$^{-/-}$ mice given intraperitoneal injection of VSV ($2×10^7$ pfu/g) for 3 d was performed by H&E staining; scale bar, 150 μm. Data of (C-E) from three independent experiments are presented as mean ± SD; ****P* < 0.001 indicates significant difference by unpaired Student's *t*-test; Data of (F) are representative results of three independent experiments.

Han Y. et al, Fig. S5

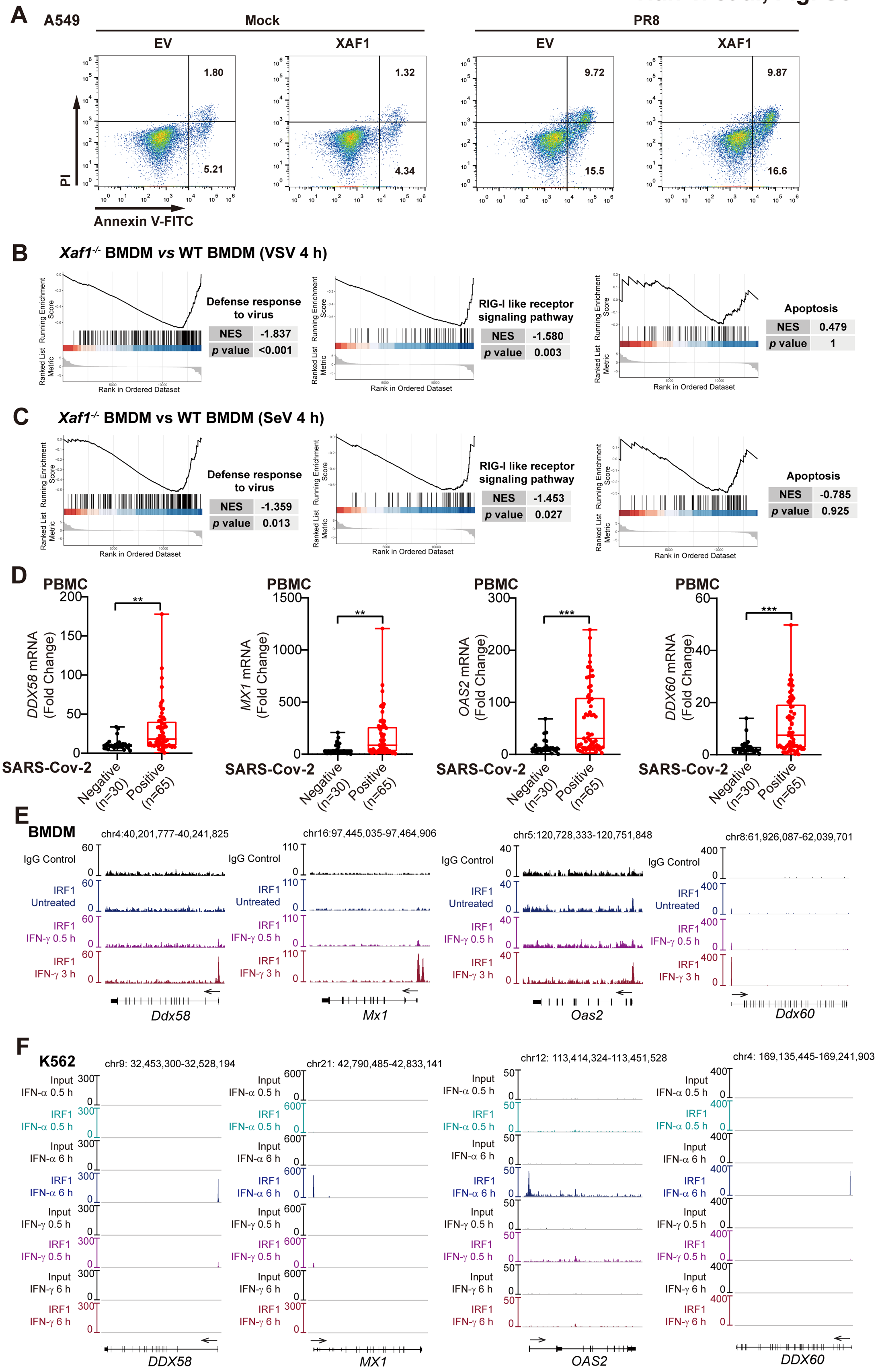

Fig. S5. Attenuated induction of IRF1 target genes in the *Xaf1*$^{-/-}$ macrophages. (A) Flow cytometry analysis of cell apoptosis in the A549 cells transfected with empty vector (EV) or XAF1 plasmid, followed by PR8 infection (MOI 2) for 24 h. (B and C) GSEA analysis of GO and KEGG gene sets for the *Xaf1*$^{-/-}$ BMDMs, comparing with the WT BMDMs after VSV-infection (B) and SeV-infection (C). NES< -1 and $P < 0.05$ indicate lower expression of genes related with Defense response to virus and RIG-I like receptor signaling pathway. No significance in Apoptosis signaling pathway between the WT and *Xaf1*$^{-/-}$ cells during viral infection. (D) The PBMCs were from healthy controls (n=30) and SARS-CoV-2-positive COVID-19 patients (n=65). Indicated genes (*DDX58*, *MX1*, *OAS2*, *DDX60*) mRNA levels of these samples were analyzed by RNA-seq. (E) BMDMs were treated with 400 U/ml IFN-γ for the indicated time points, IRF1 ChIP-Seq data were analyzed, and the IRF1 binding regions in the indicated promoters are shown. The IRF1 ChIP-Seq raw data were downloaded from GEO (accession no. GSE77886). (F) K562 cells were treated with IFN-α or IFN-γ for the indicated time points, IRF1 ChIP-Seq data were analyzed, and the IRF1 binding regions in the indicated promoters are shown. The IRF1 ChIP-Seq raw data were downloaded from GEO (accession no. GSE31477). Data of (D) from three independent experiments are presented as mean ± SD; $^{**}P < 0.01$, $^{***}P < 0.001$ by unpaired Student's *t*-test. Data of (A, F) are representative results of three independent experiments.

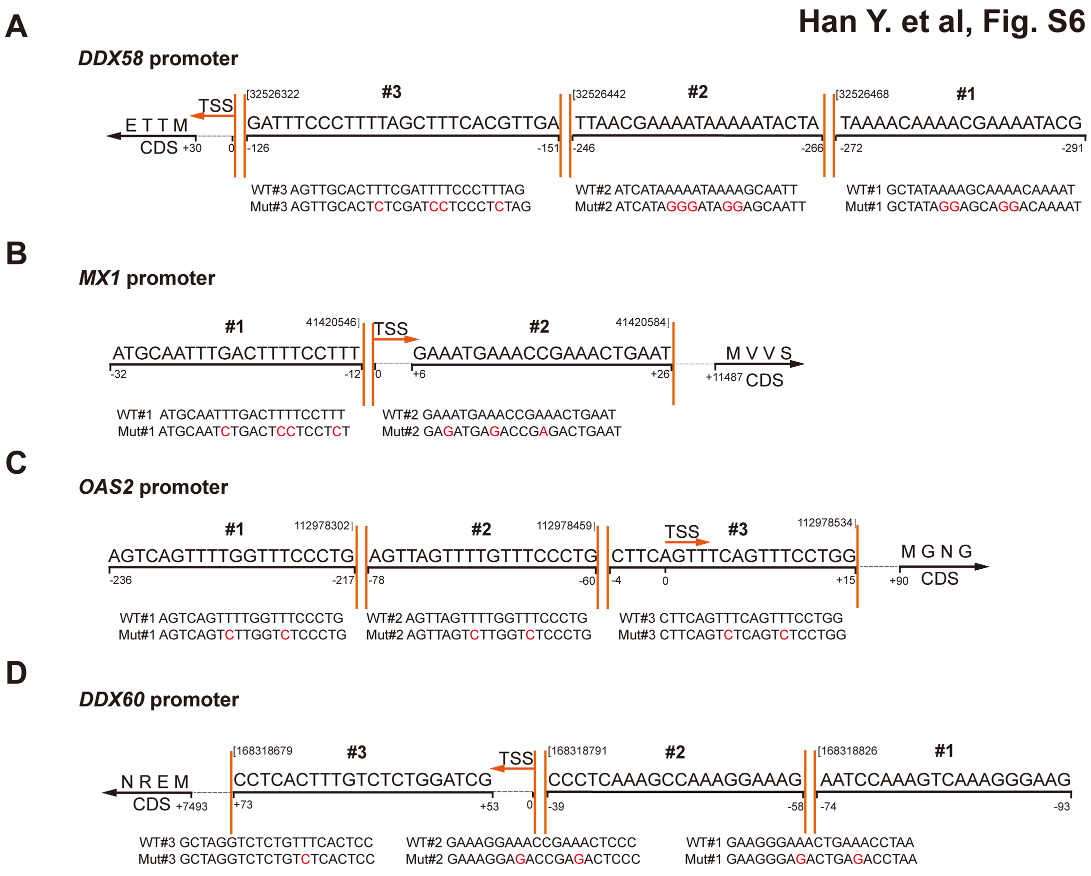

Fig. 6. Potential IRF1 binding sites in the promoters of IRF1 targeted gene. (A) The model of potential IRF1 binding sites in the *DDX58* promoter. (B) The model of potential IRF1 binding sites in the *MX1* promoter. (C) The model of potential IRF1 binding sites in the *OAS2* promoter. (D) The model of potential IRF1 binding sites in the *DDX60* promoter.

Han Y. et al, Fig. S7

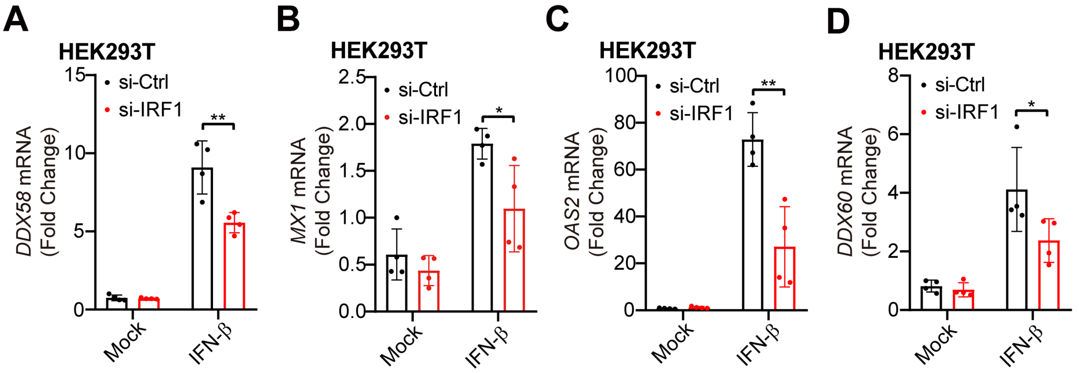

Fig. S7. Attenuated induction of IRF1 target genes after IRF1 knockdown. (A-D) qRT-PCR analysis of *DDX58* (A), *MX1* (B), *OAS2* (C), and *DDX60* (D) mRNA levels in HEK293T cells pretreated for 48 h with IRF1 siRNA, followed by IFN-β (100 U/ml) treatment for 6 h. All data from three independent experiments are presented as mean ± SD; **P* < 0.05 and ***P* < 0.01 indicate significant differences by unpaired Student's *t*-test.
